# Supplementary material for: Grafting or pruning in the animal tree: lateral gene transfer and gene loss?
Source: BMC Genomics. 2018 Jun 18;19:470. doi: 10.1186/s12864-018-4832-5 (PMC6006793; doi:10.1186/s12864-018-4832-5)
Supplement: Supplementary file 1 — EggNOG tree for COG2988 as of March 2, 2018. (PDF 229 kb) [file 12864_2018_4832_MOESM1_ESM.pdf]

Found 1 matches in 0.01 seconds (1KB)

Query OG

COG2988

✖

Add target taxa...

COG2988

All organisms

361 proteins | 283 species

E Amino acid transport and metabolism

Transforms N(2)-succinylglutamate into succinate and glutamate (By similarity)

Ortholog	Organism
ENSMGAG00000006753	*Meleagris gallopavo*
RS9916_34942	*Synechococcus sp. RS9916*
ASTE	*Burkholderia sp. CCGE1003*
ASTE	*Alteromonas sp. SN2*
ASTE	*Alteromonas macleodii str. Deep ecotype*
ASTE	*Vibrio cholerae O395*
PCC7424_3769	*Cyanothece sp. PCC 7424*
ASPA	*Prochlorococcus marinus str. AS9601*

353 more...

Fine-grained Orthologs | Orthologous Group | Taxonomic Profile | Functional Profile | Alignment | Phylogenetic Tree | Download ▾

×

Flat tree | PFAM domains | SMART domains | Aligned blocks

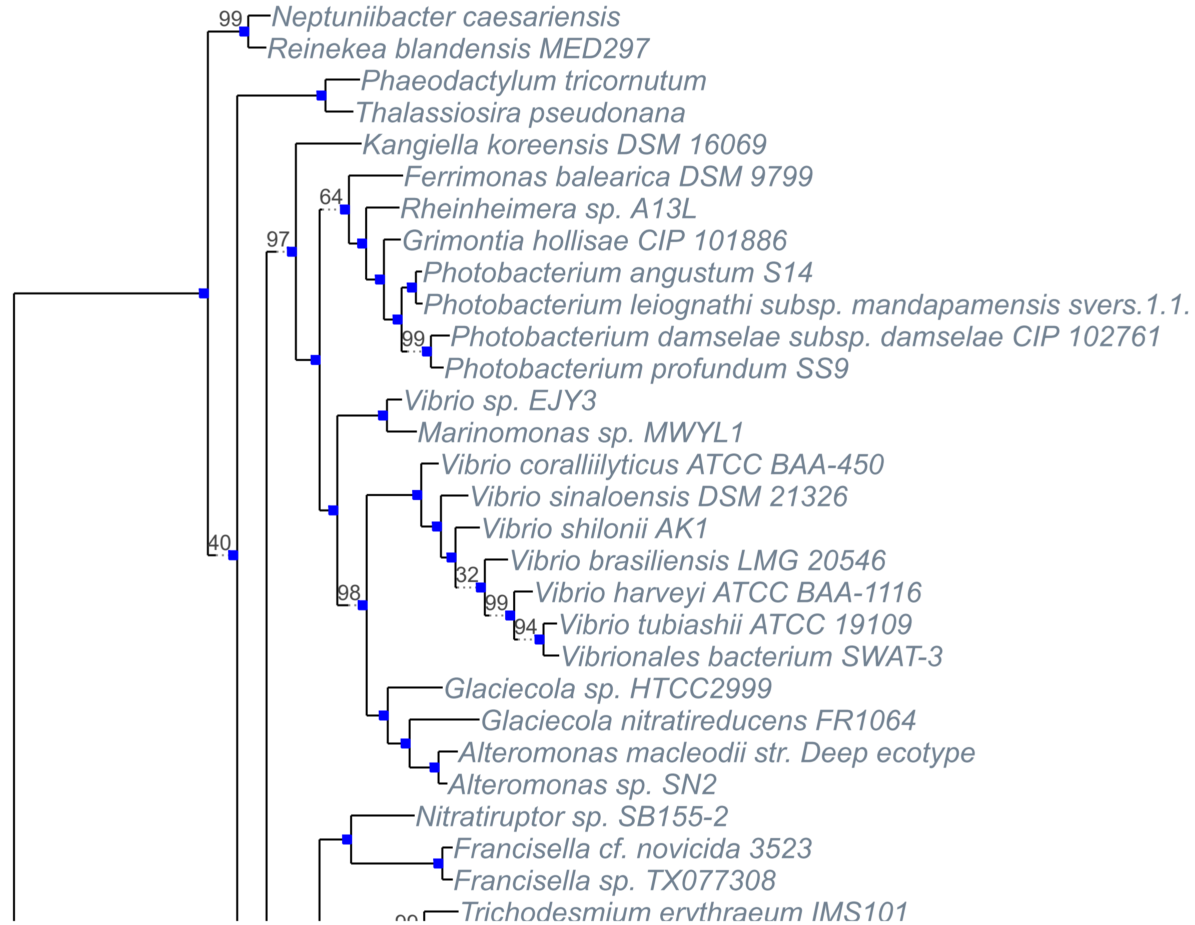

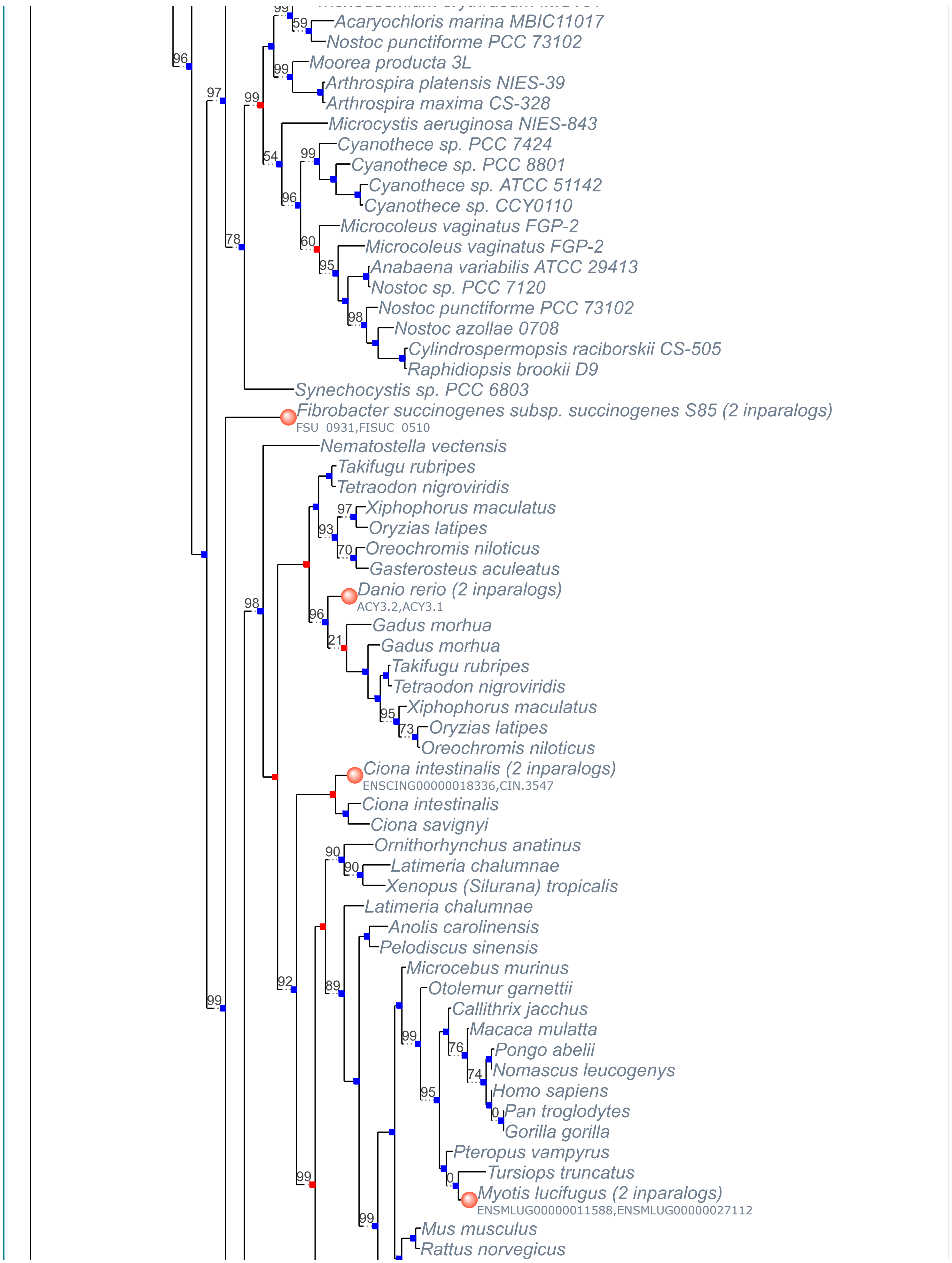
Acaryochloris marina MBIC11017
Nostoc punctiforme PCC 73102
Moorea producta 3L
Arthrospira platensis NIES-39
Arthrospira maxima CS-328
Microcystis aeruginosa NIES-843
Cyanothece sp. PCC 7424
Cyanothece sp. PCC 8801
Cyanothece sp. ATCC 51142
Cyanothece sp. CCY0110
Microcoleus vaginatus FGP-2
Microcoleus vaginatus FGP-2
Anabaena variabilis ATCC 29413
Nostoc sp. PCC 7120
Nostoc punctiforme PCC 73102
Nostoc azollae 0708
Cylindrospermopsis raciborskii CS-505
Raphidiopsis brookii D9
Synechocystis sp. PCC 6803
Fibrobacter succinogenes subsp. succinogenes S85 (2 inparalogs)
FSU_0931,FISUC_0510
Nematostella vectensis
Takifugu rubripes
Tetraodon nigroviridis
Xiphophorus maculatus
Oryzias latipes
Oreochromis niloticus
Gasterosteus aculeatus
Danio rerio (2 inparalogs)
ACY3.2,ACY3.1
Gadus morhua
Gadus morhua
Takifugu rubripes
Tetraodon nigroviridis
Xiphophorus maculatus
Oryzias latipes
Oreochromis niloticus
Ciona intestinalis (2 inparalogs)
ENSCING00000018336,CIN.3547
Ciona intestinalis
Ciona savignyi
Ornithorhynchus anatinus
Latimeria chalumnae
Xenopus (Silurana) tropicalis
Latimeria chalumnae
Anolis carolinensis
Pelodiscus sinensis
Microcebus murinus
Otolemur garnettii
Callithrix jacchus
Macaca mulatta
Pongo abelii
Nomascus leucogenys
Homo sapiens
Pan troglodytes
Gorilla gorilla
Pteropus vampyrus
Tursiops truncatus
Myotis lucifugus (2 inparalogs)
ENSMLUG00000011588,ENSMLUG00000027112
Mus musculus
Rattus norvegicus
96
97
99
59
99
99
54
99
96
78
60
95
98
98
97
93
70
96
21
95
73
90
90
92
89
99
99
99
76
74
95
0
0
99

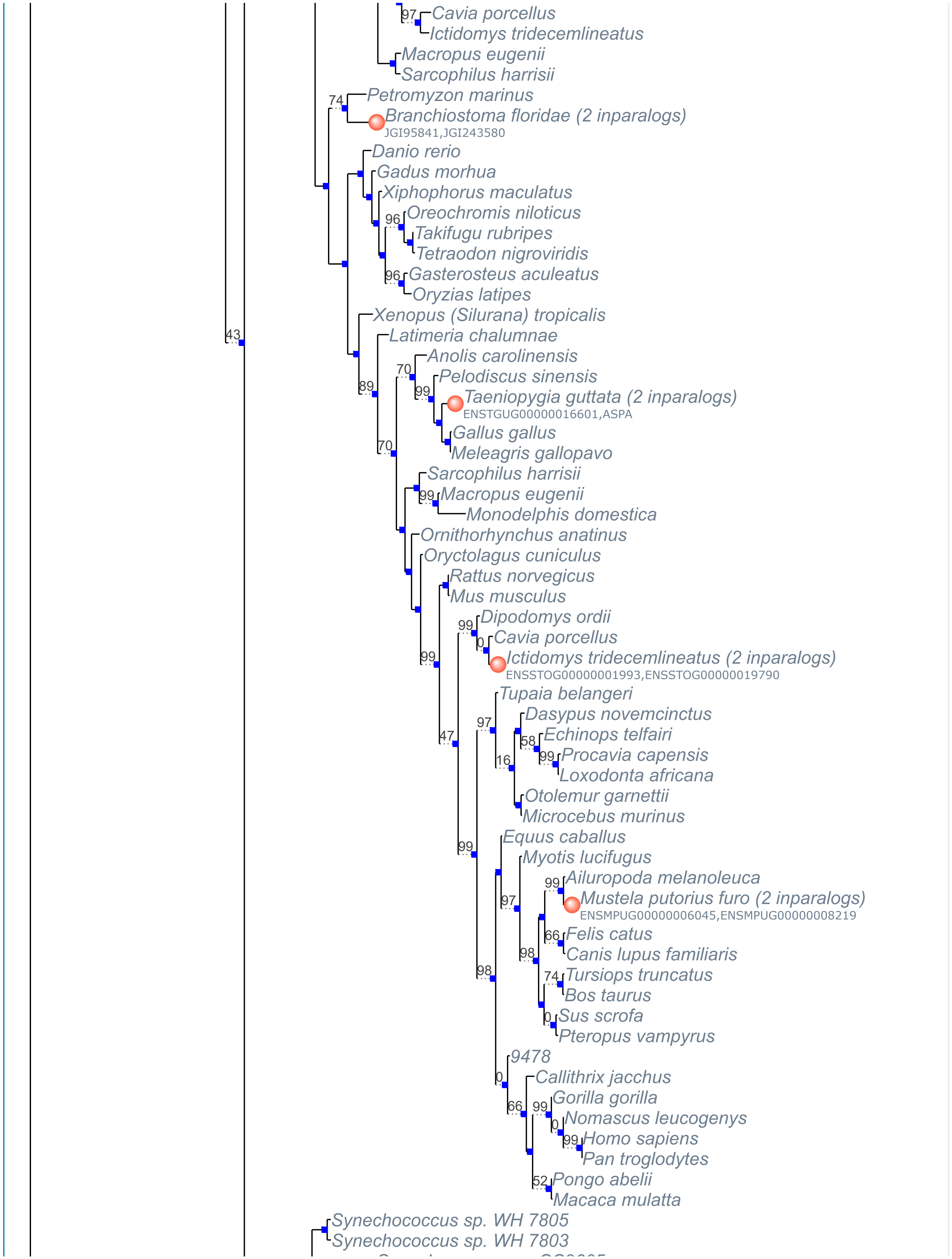
Cavia porcellus
Ictidomys tridecemlineatus
Macropus eugenii
Sarcophilus harrisii
Petromyzon marinus
Branchiostoma floridae (2 inparalogs)
JGI95841,JGI243580
Danio rerio
Gadus morhua
Xiphophorus maculatus
Oreochromis niloticus
Takifugu rubripes
Tetraodon nigroviridis
Gasterosteus aculeatus
Oryzias latipes
Xenopus (Silurana) tropicalis
Latimeria chalumnae
Anolis carolinensis
Pelodiscus sinensis
Taeniopygia guttata (2 inparalogs)
ENSTGUG00000016601,ASPA
Gallus gallus
Meleagris gallopavo
Sarcophilus harrisii
Macropus eugenii
Monodelphis domestica
Ornithorhynchus anatinus
Oryctolagus cuniculus
Rattus norvegicus
Mus musculus
Dipodomys ordii
Cavia porcellus
Ictidomys tridecemlineatus (2 inparalogs)
ENSSTOG00000001993,ENSSTOG00000019790
Tupaia belangeri
Dasypus novemcinctus
Echinops telfairi
Procavia capensis
Loxodonta africana
Otolemur garnettii
Microcebus murinus
Equus caballus
Myotis lucifugus
Ailuropoda melanoleuca
Mustela putorius furo (2 inparalogs)
ENSMPUG00000006045,ENSMPUG00000008219
Felis catus
Canis lupus familiaris
Tursiops truncatus
Bos taurus
Sus scrofa
Pteropus vampyrus
9478
Callithrix jacchus
Gorilla gorilla
Nomascus leucogenys
Homo sapiens
Pan troglodytes
Pongo abelii
Macaca mulatta
Synechococcus sp. WH 7805
Synechococcus sp. WH 7803

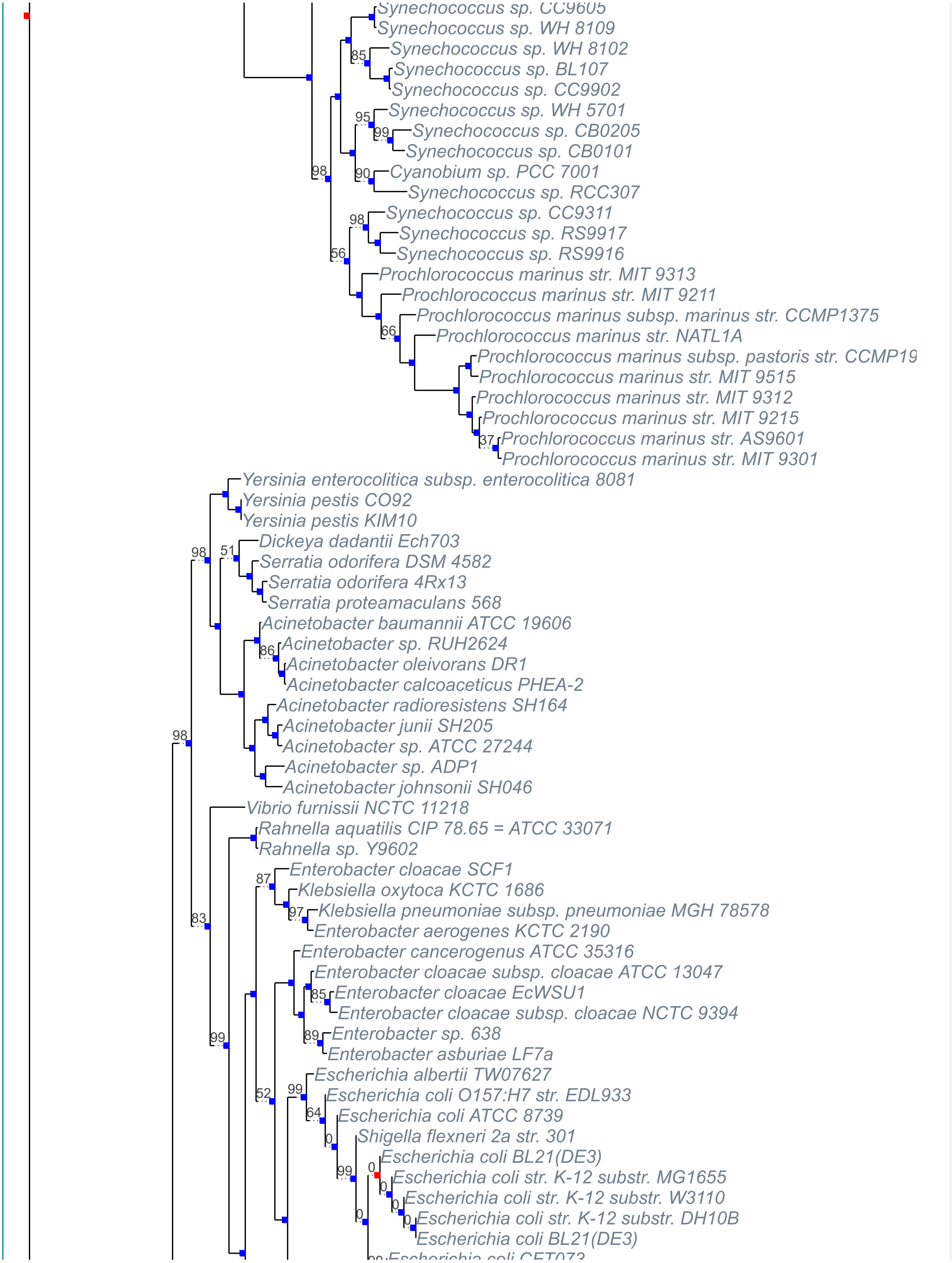
Synechococcus sp. CC9605
Synechococcus sp. WH 8109
Synechococcus sp. WH 8102
Synechococcus sp. BL107
Synechococcus sp. CC9902
Synechococcus sp. WH 5701
Synechococcus sp. CB0205
Synechococcus sp. CB0101
Cyanobium sp. PCC 7001
Synechococcus sp. RCC307
Synechococcus sp. CC9311
Synechococcus sp. RS9917
Synechococcus sp. RS9916
Prochlorococcus marinus str. MIT 9313
Prochlorococcus marinus str. MIT 9211
Prochlorococcus marinus subsp. marinus str. CCMP1375
Prochlorococcus marinus str. NATL1A
Prochlorococcus marinus subsp. pastoris str. CCMP19
Prochlorococcus marinus str. MIT 9515
Prochlorococcus marinus str. MIT 9312
Prochlorococcus marinus str. MIT 9215
Prochlorococcus marinus str. AS9601
Prochlorococcus marinus str. MIT 9301
Yersinia enterocolitica subsp. enterocolitica 8081
Yersinia pestis CO92
Yersinia pestis KIM10
Dickeya dadantii Ech703
Serratia odorifera DSM 4582
Serratia odorifera 4Rx13
Serratia proteamaculans 568
Acinetobacter baumannii ATCC 19606
Acinetobacter sp. RUH2624
Acinetobacter oleivorans DR1
Acinetobacter calcoaceticus PHEA-2
Acinetobacter radioresistens SH164
Acinetobacter junii SH205
Acinetobacter sp. ATCC 27244
Acinetobacter sp. ADP1
Acinetobacter johnsonii SH046
Vibrio furnissii NCTC 11218
Rahnella aquatilis CIP 78.65 = ATCC 33071
Rahnella sp. Y9602
Enterobacter cloacae SCF1
Klebsiella oxytoca KCTC 1686
Klebsiella pneumoniae subsp. pneumoniae MGH 78578
Enterobacter aerogenes KCTC 2190
Enterobacter cancerogenus ATCC 35316
Enterobacter cloacae subsp. cloacae ATCC 13047
Enterobacter cloacae EcWSU1
Enterobacter cloacae subsp. cloacae NCTC 9394
Enterobacter sp. 638
Enterobacter asburiae LF7a
Escherichia albertii TW07627
Escherichia coli O157:H7 str. EDL933
Escherichia coli ATCC 8739
Shigella flexneri 2a str. 301
Escherichia coli BL21(DE3)
Escherichia coli str. K-12 substr. MG1655
Escherichia coli str. K-12 substr. W3110
Escherichia coli str. K-12 substr. DH10B
Escherichia coli BL21(DE3)

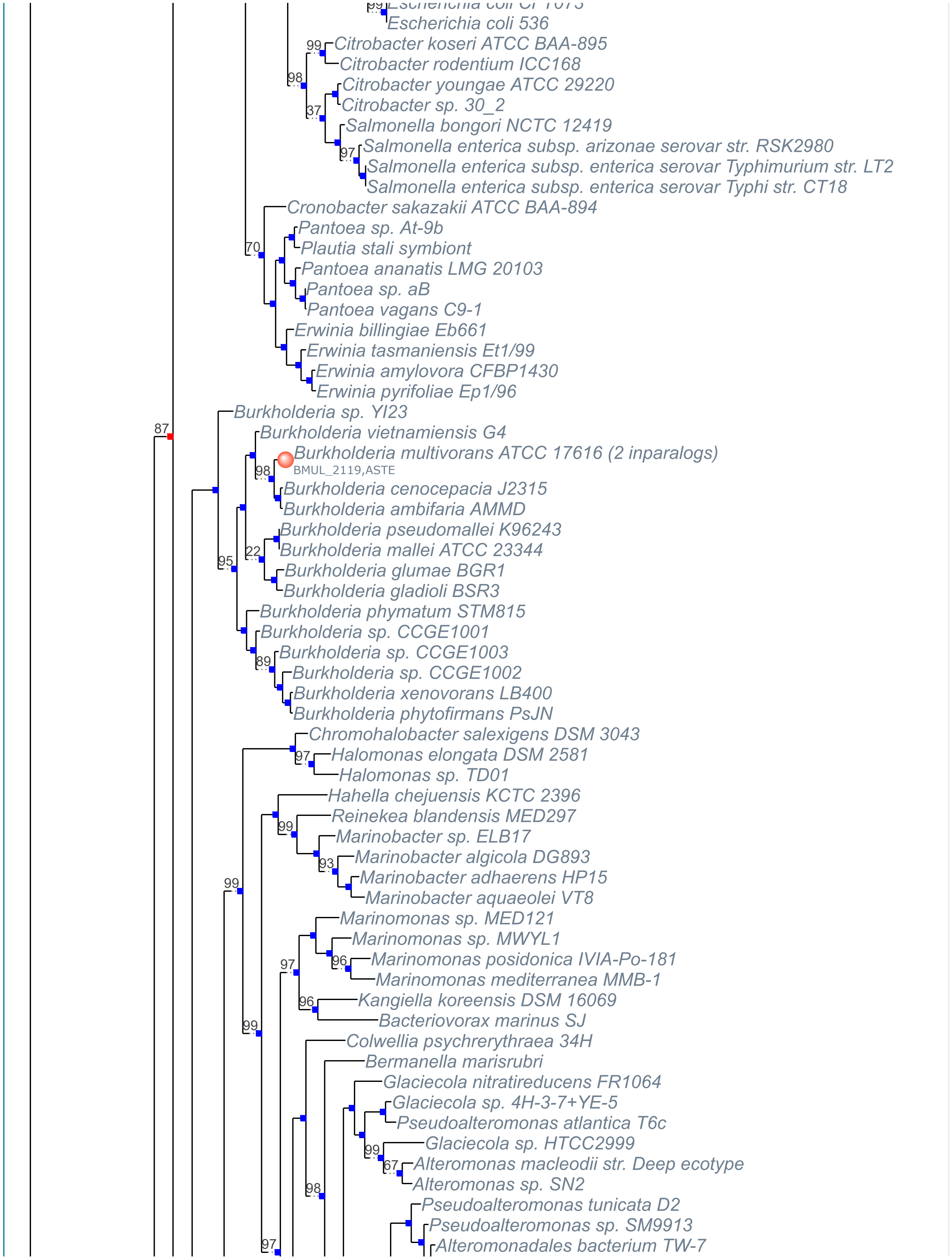
Escherichia coli 536
Citrobacter koseri ATCC BAA-895
Citrobacter rodentium ICC168
Citrobacter youngae ATCC 29220
Citrobacter sp. 30_2
Salmonella bongori NCTC 12419
Salmonella enterica subsp. arizonae serovar str. RSK2980
Salmonella enterica subsp. enterica serovar Typhimurium str. LT2
Salmonella enterica subsp. enterica serovar Typhi str. CT18
Cronobacter sakazakii ATCC BAA-894
Pantoea sp. At-9b
Plautia stali symbiont
Pantoea ananatis LMG 20103
Pantoea sp. aB
Pantoea vagans C9-1
Erwinia billingiae Eb661
Erwinia tasmaniensis Et1/99
Erwinia amylovora CFBP1430
Erwinia pyrifoliae Ep1/96
Burkholderia sp. YI23
Burkholderia vietnamiensis G4
Burkholderia multivorans ATCC 17616 (2 inparalogs)
BMUL_2119,ASTE
Burkholderia cenocepacia J2315
Burkholderia ambifaria AMMD
Burkholderia pseudomallei K96243
Burkholderia mallei ATCC 23344
Burkholderia glumae BGR1
Burkholderia gladioli BSR3
Burkholderia phymatum STM815
Burkholderia sp. CCGE1001
Burkholderia sp. CCGE1003
Burkholderia sp. CCGE1002
Burkholderia xenovorans LB400
Burkholderia phytofirmans PsJN
Chromohalobacter salexigens DSM 3043
Halomonas elongata DSM 2581
Halomonas sp. TD01
Hahella chejuensis KCTC 2396
Reinekea blandensis MED297
Marinobacter sp. ELB17
Marinobacter algicola DG893
Marinobacter adhaerens HP15
Marinobacter aquaeolei VT8
Marinomonas sp. MED121
Marinomonas sp. MWYL1
Marinomonas posidonica IVIA-Po-181
Marinomonas mediterranea MMB-1
Kangiella koreensis DSM 16069
Bacteriovorax marinus SJ
Colwellia psychrerythraea 34H
Bermanella marisrubri
Glaciecola nitratireducens FR1064
Glaciecola sp. 4H-3-7+YE-5
Pseudoalteromonas atlantica T6c
Glaciecola sp. HTCC2999
Alteromonas macleodii str. Deep ecotype
Alteromonas sp. SN2
Pseudoalteromonas tunicata D2
Pseudoalteromonas sp. SM9913
Alteromonadales bacterium TW-7

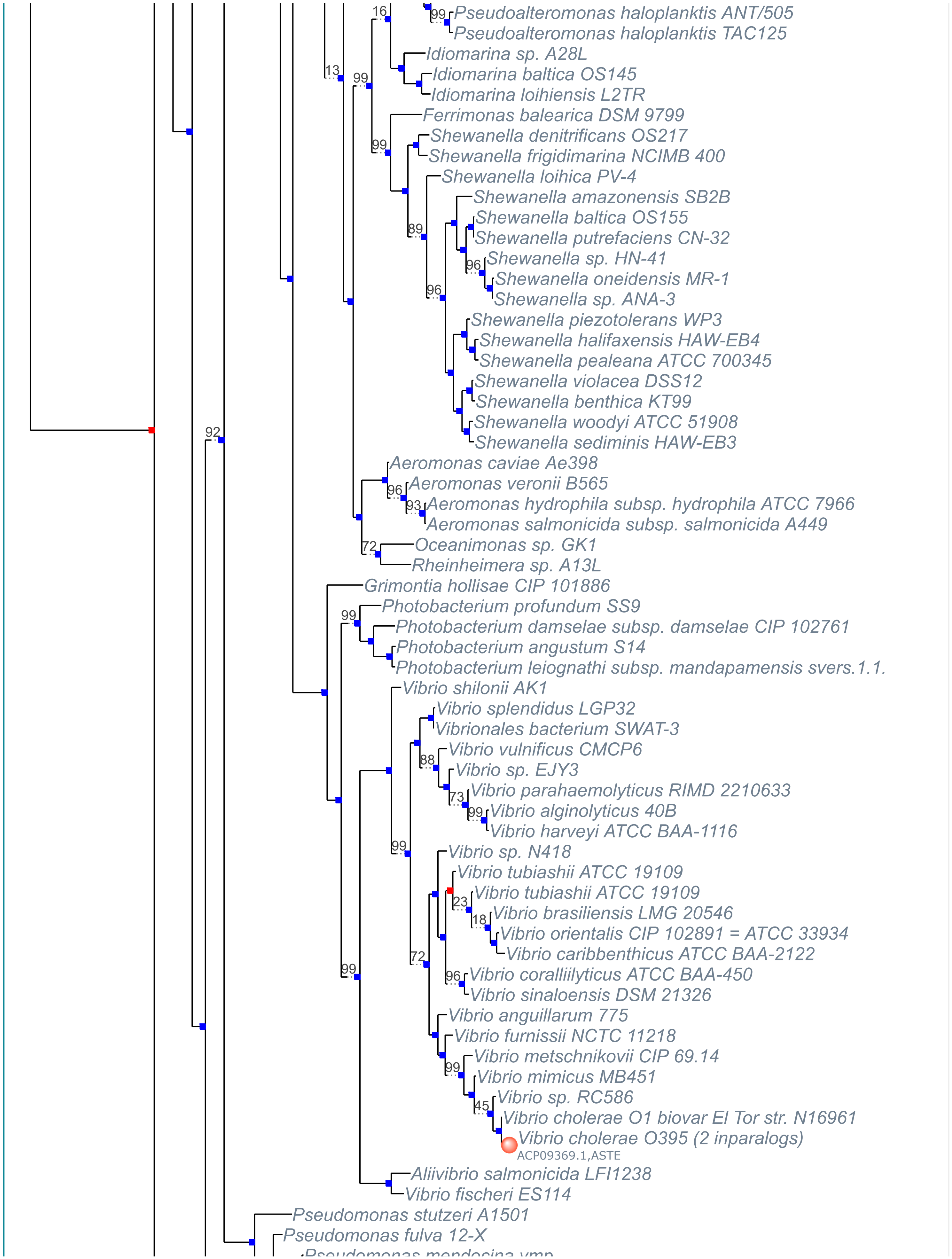
Pseudoalteromonas haloplanktis ANT/505
Pseudoalteromonas haloplanktis TAC125
Idiomarina sp. A28L
Idiomarina baltica OS145
Idiomarina loihiensis L2TR
Ferrimonas balearica DSM 9799
Shewanella denitrificans OS217
Shewanella frigidimarina NCIMB 400
Shewanella loihica PV-4
Shewanella amazonensis SB2B
Shewanella baltica OS155
Shewanella putrefaciens CN-32
Shewanella sp. HN-41
Shewanella oneidensis MR-1
Shewanella sp. ANA-3
Shewanella piezotolerans WP3
Shewanella halifaxensis HAW-EB4
Shewanella pealeana ATCC 700345
Shewanella violacea DSS12
Shewanella benthica KT99
Shewanella woodyi ATCC 51908
Shewanella sediminis HAW-EB3
Aeromonas caviae Ae398
Aeromonas veronii B565
Aeromonas hydrophila subsp. hydrophila ATCC 7966
Aeromonas salmonicida subsp. salmonicida A449
Oceanimonas sp. GK1
Rheinheimera sp. A13L
Grimontia hollisae CIP 101886
Photobacterium profundum SS9
Photobacterium damselae subsp. damselae CIP 102761
Photobacterium angustum S14
Photobacterium leiognathi subsp. mandapamensis svers.1.1.
Vibrio shilonii AK1
Vibrio splendidus LGP32
Vibrionales bacterium SWAT-3
Vibrio vulnificus CMCP6
Vibrio sp. EJY3
Vibrio parahaemolyticus RIMD 2210633
Vibrio alginolyticus 40B
Vibrio harveyi ATCC BAA-1116
Vibrio sp. N418
Vibrio tubiashii ATCC 19109
Vibrio tubiashii ATCC 19109
Vibrio brasiliensis LMG 20546
Vibrio orientalis CIP 102891 = ATCC 33934
Vibrio caribbenthicus ATCC BAA-2122
Vibrio coralliilyticus ATCC BAA-450
Vibrio sinaloensis DSM 21326
Vibrio anguillarum 775
Vibrio furnissii NCTC 11218
Vibrio metschnikovii CIP 69.14
Vibrio mimicus MB451
Vibrio sp. RC586
Vibrio cholerae O1 biovar El Tor str. N16961
Vibrio cholerae O395 (2 inparalogs)
ACP09369.1,ASTE
Aliivibrio salmonicida LFI1238
Vibrio fischeri ES114
Pseudomonas stutzeri A1501
Pseudomonas fulva 12-X

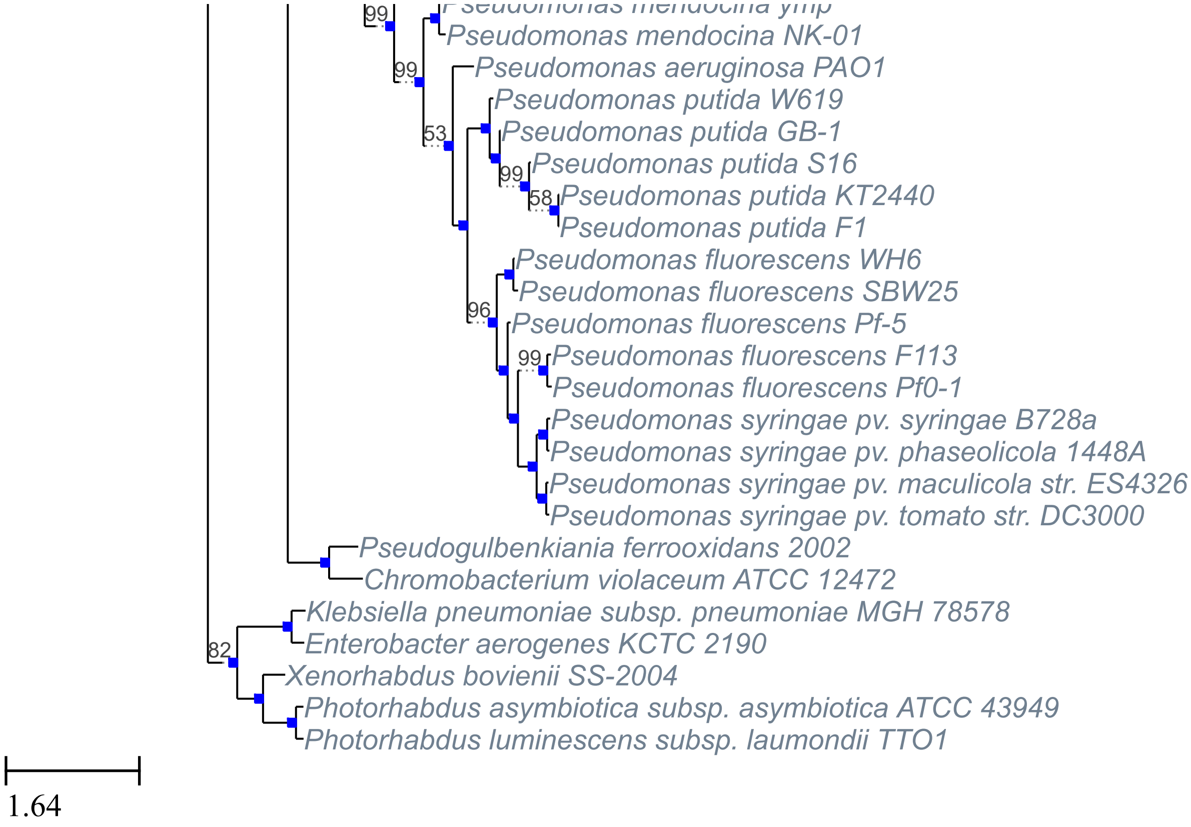

Computational Biology group (http://www.bork.embl.de) - EMBL, Heidelberg. (http://embl.de)
© 2016 The EggNOG database Team - v4.5 ⭡()
