# Supplementary material for: Grafting or pruning in the animal tree: lateral gene transfer and gene loss?
Source: BMC Genomics. 2018 Jun 18;19:470. doi: 10.1186/s12864-018-4832-5 (PMC6006793; doi:10.1186/s12864-018-4832-5)
Supplement: Supplementary file 2 — TBLASTN search results against NR of A. pisum sequence from PFAM (J9KVH7) with homology to ASPA/ACY3 homologues. (PDF 264 kb) [file 12864_2018_4832_MOESM2_ESM.pdf]

BLAST ® » tblastn » RID-9JPWJAZR014

BLAST Results

Questions/comments

Job title: Protein Sequence (333 letters)

**RID**	9JPWJAZR014 (Expires on 03-03 21:11 pm)		
**Query ID**	lcl|Query_218831	**Database Name**	nr
**Description**	None	**Description**	Nucleotide collection (nt)
**Molecule type**	amino acid	**Program**	TBLASTN 2.8.0+
**Query Length**	333		

Graphic Summary

Distribution of the top 183 Blast Hits on 100 subject sequences

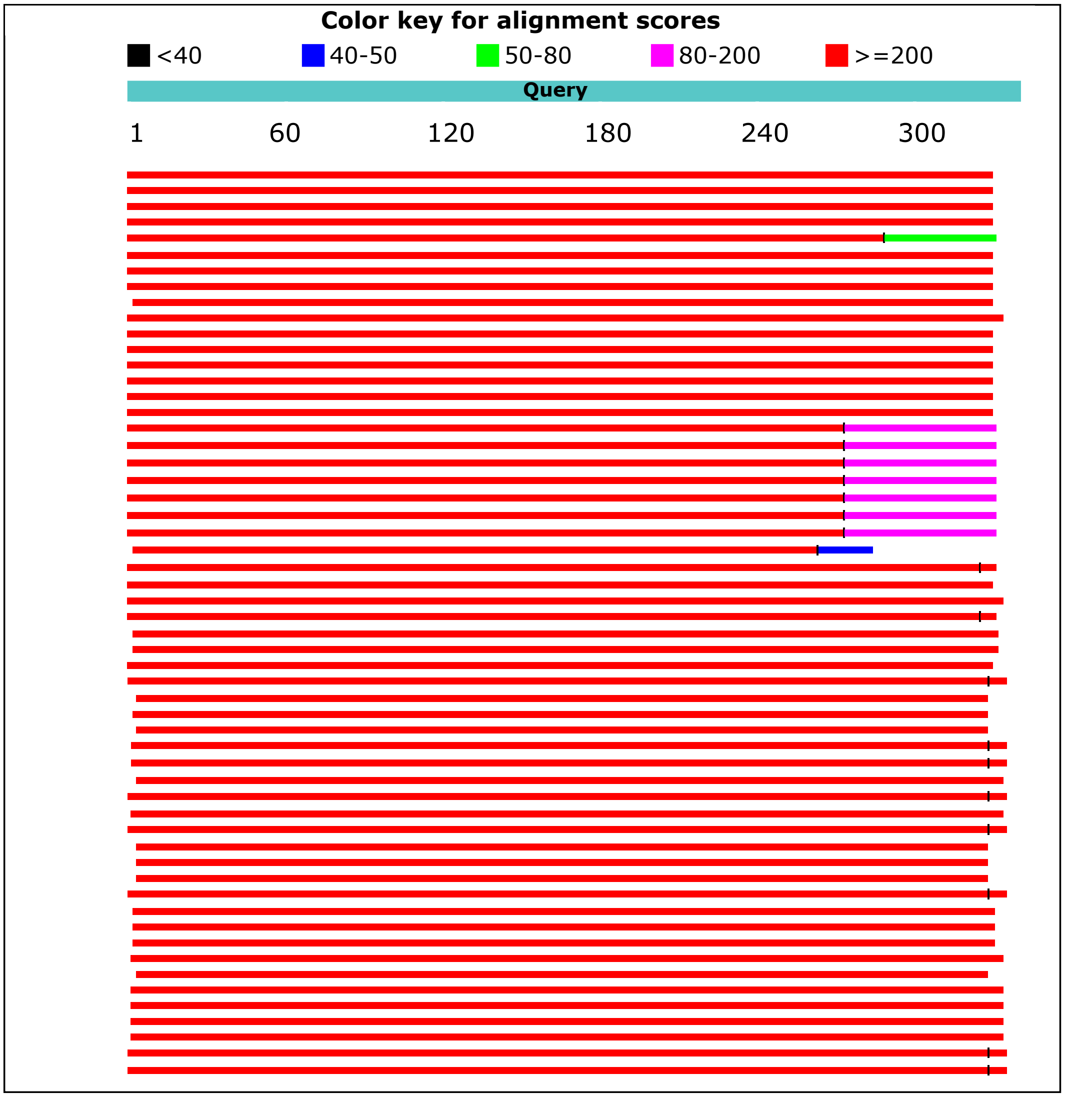

Descriptions

Sequences producing significant alignments:

Description	Max score	Total score	Query cover	E value	Ident	Accession
Pantoea rwandensis strain ND04, complete genome	667	667	98%	0.0	98%	CP009454.1
Pantoea vagans strain ND02, complete genome	649	649	98%	0.0	95%	CP011427.1
Pantoea sp. At-9b, complete genome	613	613	98%	0.0	89%	CP002433.1
Pantoea sp. PSNIH1, complete genome	597	597	98%	0.0	85%	CP009880.2
Plautia stali symbiont DNA, complete genome	535	612	98%	5e-170	90%	AP012551.1
Pantoea vagans strain FDAARGOS_160 chromosome, complete genome	530	530	98%	2e-168	69%	CP014129.2
Pantoea agglomerans strain C410P1, complete genome	527	527	98%	2e-167	69%	CP016889.1
Pantoea vagans C9-1, complete genome	522	522	98%	1e-165	68%	CP002206.1
Pluralibacter gergoviae strain FB2, complete genome	476	476	98%	1e-149	69%	CP009450.1
Erwinia billingiae strain Eb661 complete chromosome	474	474	100%	5e-149	69%	FP236843.1
Erwinia tasmaniensis strain ET1/99 complete chromosome	456	456	98%	2e-142	67%	CU468135.1
Erwinia pyrifoliae DSM 12163 complete genome, culture collection DSM:12163	449	449	98%	4e-140	66%	FN392235.1
Erwinia pyrifoliae strain Ep1/96 complete chromosome	449	449	98%	4e-140	66%	FP236842.1
Erwinia pyrifoliae strain EpK1/15 chromosome, complete genome	449	449	98%	4e-140	66%	CP023567.1
Erwinia sp. Ejp617, complete genome	447	447	98%	2e-139	66%	CP002124.1
Gibbsiella quercinecans strain FRB97, complete genome	439	439	98%	1e-136	64%	CP014136.1
Pantoea ananatis AJ13355 DNA, complete genome	422	547	98%	1e-130	74%	AP012032.2
Pantoea ananatis strain R100, complete genome	421	550	98%	1e-130	74%	CP014207.1
Pantoea ananatis PA13, complete genome	421	550	98%	1e-130	74%	CP003085.1
Pantoea ananatis LMG 5342 main chromosome complete genome	421	550	98%	1e-130	74%	HE617160.1
Pantoea ananatis strain YJ76, complete genome	421	546	98%	2e-130	73%	CP022427.1
Pantoea ananatis strain 97-1 genome	419	548	98%	5e-130	73%	CP020943.1
Pantoea ananatis LMG 20103, complete genome	417	546	98%	6e-129	73%	CP001875.2
Pantoea stewartii subsp. stewartii DC283, complete genome	405	454	84%	6e-125	73%	CP017581.1
Burkholderia sp. YI23 chromosome 2, complete sequence	398	959	98%	2e-122	59%	CP003088.1
Burkholderia sp. RPE67 DNA, complete genome, chromosome: 2	398	956	98%	2e-122	59%	AP014577.1
Burkholderia sp. RPE64 DNA, chromosome 2, complete genome	395	951	100%	1e-121	58%	AP013059.1

Description	Max score	Total score	Query cover	E value	Ident	Accession
Burkholderia sp. KK1 chromosome II sequence	395	950	98%	2e-121	59%	CP016000.1
Tatumella citrea strain ATCC 39140, complete genome	393	618	98%	8e-121	59%	CP015581.1
Tatumella citrea strain DSM 13699, complete genome	393	618	98%	8e-121	59%	CP015579.1
Sodalis praecaptivus strain HS1, complete genome	370	370	98%	6e-113	57%	CP006569.1
Pseudomonas extremaustralis strain DSM 17835 genome assembly, chromosome: I	313	806	100%	4e-93	48%	LT629689.1
Pseudomonas putida DNA, complete genome, strain: KF715	313	313	97%	5e-93	49%	AP015029.1
Pseudomonas cremoricolorata strain ND07, complete genome	313	313	97%	6e-93	49%	CP009455.1
Pseudomonas putida H8234, complete genome	311	311	97%	2e-92	49%	CP005976.1
Pseudomonas fragi strain P121, complete genome	311	796	99%	3e-92	49%	CP013861.1
Pseudomonas sp. Lz4W chromosome, complete genome	310	796	99%	5e-92	48%	CP017432.1
Pseudomonas sp. HLS-6 chromosome, complete genome	310	310	99%	6e-92	46%	CP024478.1
Pseudomonas azotoformans strain LMG 21611 genome assembly, chromosome: I	309	665	100%	1e-91	48%	LT629702.1
Pseudomonas synxantha strain LMG 2190 genome assembly, chromosome: I	309	540	99%	1e-91	48%	LT629786.1
Pseudomonas extremorientalis strain BS2774 genome assembly, chromosome: I	309	538	100%	2e-91	48%	LT629708.1
Pseudomonas putida strain 1A00316, complete genome	308	308	97%	2e-91	48%	CP014343.1
Pseudomonas mosselii strain PtA1 chromosome, complete genome	308	308	97%	2e-91	48%	CP024159.1
Pseudomonas mosselii strain BS011 chromosome, complete genome	308	308	97%	2e-91	48%	CP023299.1
Pseudomonas palleroniana strain MAB3 chromosome	308	542	100%	3e-91	48%	CP025494.1
Pseudomonas sp. PONIH3 chromosome, complete genome	308	546	98%	4e-91	48%	CP026386.1
Pseudomonas mosselii SJ10, complete genome	307	547	98%	7e-91	47%	CP009365.1
Pseudomonas sp. CCOS 191 genome assembly Pseudomonas sp. strain CCOS 191, chromosome : I	307	545	98%	7e-91	47%	LN847264.1
Pseudomonas sp. FDAARGOS_380 chromosome, complete genome	307	543	99%	7e-91	49%	CP023969.1
Pseudomonas sp. 02C 26 chromosome	307	307	97%	8e-91	49%	CP025262.1
Pseudomonas cichorii JBC1, complete genome	307	546	99%	1e-90	47%	CP007039.1
Pseudomonas fluorescens strain KENGFT3 genome	307	536	99%	1e-90	48%	CP014868.1
Pseudomonas fluorescens strain LBUM223, complete genome	307	536	99%	1e-90	48%	CP011117.1
Pseudomonas libanensis strain BS2975 genome assembly, chromosome: I	307	541	99%	1e-90	48%	LT629699.1

Description	Max score	Total score	Query cover	E value	Ident	Accession
Pseudomonas azotoformans strain S4, complete genome	306	900	100%	1e-90	48%	CP014546.1
Pseudomonas fluorescens strain L228, complete genome	306	907	100%	1e-90	47%	CP015639.1
Burkholderia ambifaria AMMD chromosome 2, complete sequence	306	346	98%	2e-90	47%	CP009799.1
Burkholderia ambifaria AMMD chromosome 2, complete sequence	306	346	98%	2e-90	47%	CP000441.1
Burkholderia glumae LMG 2196 = ATCC 33617 chromosome II, complete sequence	305	305	98%	3e-90	48%	CP009434.1
Burkholderia glumae BGR1 chromosome 2, complete sequence	305	305	98%	3e-90	48%	CP001504.2
Pseudomonas sp. VLB120, complete genome	305	563	99%	3e-90	48%	CP003961.1
Pseudomonas sp. SWI6 chromosome, complete genome	305	563	99%	3e-90	48%	CP026676.1
Pseudomonas sp. SWI44 chromosome, complete genome	305	563	99%	3e-90	48%	CP026674.1
Pseudomonas fluorescens strain ATCC 13525 genome assembly, chromosome: I	305	652	100%	4e-90	48%	LT907842.1
Burkholderia ambifaria MC40-6 chromosome 2, complete sequence	305	560	98%	4e-90	48%	CP001026.1
Burkholderia sp. PAMC 26561 chromosome 2, complete sequence	305	573	99%	5e-90	46%	CP014307.1
Burkholderia sp. PAMC 28687 strain PAMC28687 chromosome 3, complete sequence	305	571	99%	5e-90	46%	CP014507.1
Paraburkholderia caribensis strain MWAP64 chromosome 2, complete sequence	305	566	98%	5e-90	48%	CP013103.1
Paraburkholderia caribensis strain DSM 13236 chromosome 2, complete sequence	305	566	98%	5e-90	48%	CP026102.1
Paraburkholderia caribensis strain Bcrs1W chromosome 2, complete sequence	305	567	98%	5e-90	48%	CP013348.1
Pseudomonas putida S13.1.2, complete genome	305	305	97%	6e-90	48%	CP010979.1
Paraburkholderia terrae strain DSM 17804 chromosome 2, complete sequence	305	575	98%	6e-90	48%	CP026112.1
Pseudomonas orientalis strain F9, complete genome	304	781	100%	7e-90	48%	CP018049.1
Pseudomonas entomophila str. L48 chromosome,complete sequence	304	556	98%	8e-90	48%	CT573326.1
Pseudomonas putida strain IEC33019, complete genome	304	304	97%	9e-90	48%	CP016634.1
Paraburkholderia hospita strain DSM 17164 chromosome 2, complete sequence	304	572	98%	9e-90	47%	CP026106.1
Pseudomonas tolaasii strain 2192T genome	304	894	100%	1e-89	48%	CP020369.1
Burkholderia plantarii strain ATCC 43733 chromosome 2, complete sequence	303	303	98%	1e-89	47%	CP007213.1
Pseudomonas sp. NC02 chromosome, complete genome	303	902	100%	1e-89	47%	CP025624.1
Pseudomonas putida NBRC 14164 DNA, complete genome	303	303	97%	1e-89	48%	AP013070.1
Pseudomonas putida strain PP112420, complete genome	303	303	97%	2e-89	48%	CP017073.1

Description	Max score	Total score	Query cover	E value	Ident	Accession
Pseudomonas putida GB-1, complete genome	303	303	97%	2e-89	48%	CP000926.1
Pseudomonas syringae pv. syringae B728a, complete genome	303	542	99%	2e-89	47%	CP000075.1
Burkholderia glumae PG1 chromosome 2, complete sequence	303	303	98%	2e-89	47%	CP002581.1
Pseudomonas viridiflava strain CFBP 1590 genome assembly, chromosome: I	303	551	99%	2e-89	48%	LT855380.1
Pseudomonas trivialis strain IHBB745, complete genome	303	650	100%	3e-89	47%	CP011507.1
Pseudomonas lini strain BS3782 genome assembly, chromosome: I	302	536	100%	3e-89	47%	LT629746.1
Burkholderia sp. MSMB617WGS chromosome 2, complete sequence	302	302	98%	3e-89	48%	CP013458.1
Paraburkholderia caribensis MBA4 chromosome 2, complete sequence	302	567	98%	4e-89	47%	CP012747.1
Pseudomonas syringae pv. syringae strain Pss9097 chromosome, complete genome	302	542	99%	4e-89	47%	CP026568.1
Burkholderia sp. MSMB0852 chromosome 2, complete sequence	302	302	98%	4e-89	48%	CP013425.1
Burkholderia oklahomensis strain EO147 chromosome 2, complete sequence	302	531	100%	4e-89	48%	CP008727.1
Burkholderia oklahomensis strain 1977116029 chromosome 2, complete sequence	302	531	100%	4e-89	48%	CP013356.1
Pseudomonas sp. URMO17WK12:I11 isolate Yellow genome assembly, chromosome: 1	302	302	98%	5e-89	48%	LN865164.1
Pseudomonas fulva strain FDAARGOS_167 chromosome, complete genome	302	302	98%	5e-89	48%	CP014025.1
Pseudomonas sp. S09G 359 chromosome	302	537	100%	5e-89	47%	CP025263.1
Burkholderia sp. BDU8 chromosome 2, complete sequence	301	530	99%	6e-89	48%	CP013388.1
Pseudomonas fluorescens strain L111, complete genome	301	658	100%	6e-89	47%	CP015638.1
Pseudomonas fluorescens strain L321, complete genome	301	657	100%	6e-89	47%	CP015637.1
Enterococcus faecalis strain V583 genome	301	301	97%	6e-89	48%	CP022312.1

Alignments

Pantoea rwandensis strain ND04, complete genome
Sequence ID: **CP009454.1** Length: 4327607 Number of Matches: 1
Range 1: 2084983 to 2085969

Score	Expect	Method	Identities	Positives	Gaps	Frame
667 bits(1721)	0.0()	Compositional matrix adjust.	323/329(98%)	327/329(99%)	0/329(0%)	-3

Features:

```
Query  1        SQQRVYIQAALHGDELPGMAVAWYLKHKLLALESAGQLKSKITLVPVANPLAMGQHWHGS  60
                SQQRVYIQAALHGDELPGMAVAWYLKHKLLALESAGQLKSKITLVPVANPLAMGQHWHGS
Sbjct  2085969  SQQRVYIQAALHGDELPGMAVAWYLKHKLLALESAGQLKSKITLVPVANPLAMGQHWHGS  2085790

Query  61       HLGRFHTLSGQDFNRRFPALGDTLAEELAGSLTQSEYENKRLIRDAIDRHYRDRVARTEL  120
                HLGRFHTLSGQDFNRRFPALGDTLAEELAGSLTQSEYENKRLIRDAIDRHYRDR+ARTEL
Sbjct  2085789  HLGRFHTLSGQDFNRRFPALGDTLAEELAGSLTQSEYENKRLIRDAIDRHYRDRIARTEL  2085610

Query  121      DAQRFTLMRMASQADLMIDLHCDWDALPHLYTTPHAWQDIEPLARWLGSEVQLLAQISGG  180
```

```
                DAQRFTLMRMASQADLMIDLHCDWDALPHLYTTPHAWQDIEPLARWLGSEVQLLAQISGG
Sbjct  2085609  DAQRFTLMRMASQADLMIDLHCDWDALPHLYTTPHAWQDIEPLARWLGSEVQLLAQISGG  2085430

Query  181      EPFDEACCEPWLTLAERFGGEYPMPRGLLPVTLELRGVADVSPGQAEKDADAIINALIEG  240
                EPFDEACCEPWLTLAERFGGEYPMPRGLLPVTLELRGVADVSP QAEKDADAIINALIEG
Sbjct  2085429  EPFDEACCEPWLTLAERFGGEYPMPRGLLPVTLELRGVADVSPQQAEKDADAIINALIEG  2085250

Query  241      GYIAGDVGESPALINPATPLAGCEYIHAPHSGMLLNRRELGEWIKPGEVVAEIVDPITDQ  300
                GYIAG+ GESPALINPATPLAGCEYIHAPHSG+LLNRRELGEWIKPGEVVAEIVDPITDQ
Sbjct  2085249  GYIAGETGESPALINPATPLAGCEYIHAPHSGLLLNRRELGEWIKPGEVVAEIVDPITDQ  2085070

Query  301      VTPLVAEFGGVLYARNLMKFATAGMLVVK  329
                VTPLVAEFGGVLYARNLMKFATAGMLVV+
Sbjct  2085069  VTPLVAEFGGVLYARNLMKFATAGMLVVR  2084983
```

Pantoea vagans strain ND02, complete genome

Sequence ID: **CP011427.1** Length: 4313264 Number of Matches: 1

Range 1: 1336977 to 1337963

Score	Expect	Method	Identities	Positives	Gaps	Frame
649 bits(1673)	0.0()	Compositional matrix adjust.	312/329(95%)	323/329(98%)	0/329(0%)	-1

Features:

```
Query  1        SQQRVYIQAALHGDELPGMAVAWYLKHKLLALESAGQLKSKITLVPVANPLAMGQHWHGS  60
                SQQRVYIQAALHGDELPGMAVAWYLKHKLLALESAGQLKSKITLVPVANPLAMGQHWHGS
Sbjct  1337963  SQQRVYIQAALHGDELPGMAVAWYLKHKLLALESAGQLKSKITLVPVANPLAMGQHWHGS  1337784

Query  61       HLGRFHTLSGQDFNRRFPALGDTLAEELAGSLTQSEYENKRLIRDAIDRHYRDRVARTEL  120
                HLGRFHTLSGQDFNRRFPALG+TLA ELA SLTQSEYENKRLIR+AIDRHYRDR+A+TEL
Sbjct  1337783  HLGRFHTLSGQDFNRRFPALGETLAAELADSLTQSEYENKRLIREAIDRHYRDRIAKTEL  1337604

Query  121      DAQRFTLMRMASQADLMIDLHCDWDALPHLYTTPHAWQDIEPLARWLGSEVQLLAQISGG  180
                D+QRFTLMRMASQADLMIDLHCDWDALPHLYTTPHAWQDIEPLARWLGSEVQLLAQISGG
Sbjct  1337603  DSQRFTLMRMASQADLMIDLHCDWDALPHLYTTPHAWQDIEPLARWLGSEVQLLAQISGG  1337424

Query  181      EPFDEACCEPWLTLAERFGGEYPMPRGLLPVTLELRGVADVSPGQAEKDADAIINALIEG  240
                EPFDEACCEPWLTLAERFG +YPMPRGLLPVTLELRGVADV P QAEKDADAIINALIEG
Sbjct  1337423  EPFDEACCEPWLTLAERFGDKYPMPRGLLPVTLELRGVADVEPEQAEKDADAIINALIEG  1337244

Query  241      GYIAGDVGESPALINPATPLAGCEYIHAPHSGMLLNRRELGEWIKPGEVVAEIVDPITDQ  300
                GYIAG++GESPALINPATPLAGCEYIHAPHSG+LLNRR+LGEWIKPGEVVAEIVDPITDQ
Sbjct  1337243  GYIAGELGESPALINPATPLAGCEYIHAPHSGLLLNRRKLGEWIKPGEVVAEIVDPITDQ  1337064

Query  301      VTPLVAEFGGVLYARNLMKFATAGMLVVK  329
                VTPL AEFGGVLYARNLMKFATAGMLVV+
Sbjct  1337063  VTPLFAEFGGVLYARNLMKFATAGMLVVR  1336977
```

Pantoea sp. At-9b, complete genome

Sequence ID: **CP002433.1** Length: 4368708 Number of Matches: 1

Range 1: 2809795 to 2810781

Score	Expect	Method	Identities	Positives	Gaps	Frame
613 bits(1582)	0.0()	Compositional matrix adjust.	292/329(89%)	313/329(95%)	0/329(0%)	+1

Features:

```
Query  1        SQQRVYIQAALHGDELPGMAVAWYLKHKLLALESAGQLKSKITLVPVANPLAMGQHWHGS  60
                + +RVYIQAALHGDELPGMAVAWYLK KLL LES GQLK+  TLVPVANPLA+GQHWHG+
Sbjct  2809795  AHKRVYIQAALHGDELPGMAVAWYLKQKLLNLESTGQLKASFTLVPVANPLALGQHWHGT  2809974

Query  61       HLGRFHTLSGQDFNRRFPALGDTLAEELAGSLTQSEYENKRLIRDAIDRHYRDRVARTEL  120
                HLGRFHTLSGQDFNRRFPALGDTL  ELAGSLTQSEYENKRLIRDAIDRHYRD++A+TEL
Sbjct  2809975  HLGRFHTLSGQDFNRRFPALGDTLVAELAGSLTQSEYENKRLIRDAIDRHYRDKIAKTEL  2810154

Query  121      DAQRFTLMRMASQADLMIDLHCDWDALPHLYTTPHAWQDIEPLARWLGSEVQLLAQISGG  180
                DAQRFTLMRMASQADLMIDLHCDWDA+PHLYTTPHAW DIEPLARWLGSEVQLLAQISGG
Sbjct  2810155  DAQRFTLMRMASQADLMIDLHCDWDAVPHLYTTPHAWPDIEPLARWLGSEVQLLAQISGG  2810334

Query  181      EPFDEACCEPWLTLAERFGGEYPMPRGLLPVTLELRGVADVSPGQAEKDADAIINALIEG  240
                EPFDEACCEPWLTLA R+G EYPMPRGLLPVTLELRGVADVSP QAE+DADAIINALIEG
Sbjct  2810335  EPFDEACCEPWLTLAARYGDEYPMPRGLLPVTLELRGVADVSPEQAERDADAIINALIEG  2810514

Query  241      GYIAGDVGESPALINPATPLAGCEYIHAPHSGMLLNRRELGEWIKPGEVVAEIVDPITDQ  300
                GYIAG++GESPALINPAT LAGCEYI APHSG++LNRR++GE I+ GEVVAEIVDPITDQ
Sbjct  2810515  GYIAGEIGESPALINPATALAGCEYIFAPHSGLILNRRKIGERIEAGEVVAEIVDPITDQ  2810694

Query  301      VTPLVAEFGGVLYARNLMKFATAGMLVVK  329
                VTPL+AEFGG+LYARNLMKFATAGMLVV+
Sbjct  2810695  VTPLLAEFGGILYARNLMKFATAGMLVVR  2810781
```

Pantoea sp. PSNIH1, complete genome

Sequence ID: **CP009880.2** Length: 3488376 Number of Matches: 1
Range 1: 3329203 to 3330189

Score	Expect	Method	Identities	Positives	Gaps	Frame
597 bits(1539)	0.0()	Compositional matrix adjust.	281/329(85%)	311/329(94%)	0/329(0%)	+1

Features:

```
Query  1        SQQRVYIQAALHGDELPGMAVAWYLKHKLLALESAGQLKSKITLVPVANPLAMGQHWHGS  60
                SQ+RVYIQAALHGDELPGMAVAWYLK KLLALESAG+LK+KITLVPVANPLA+GQHWHG+
Sbjct  3329203  SQKRVYIQAALHGDELPGMAVAWYLKQKLLALESAGKLKAKITLVPVANPLALGQHWHGT  3329382

Query  61       HLGRFHTLSGQDFNRRFPALGDTLAEELAGSLTQSEYENKRLIRDAIDRHYRDRVARTEL  120
                HLGRFHTLSGQDFNRRFPALG+ LA ELAGSLTQSEYEN+RLIRDAID +YRD++ARTEL
Sbjct  3329383  HLGRFHTLSGQDFNRRFPALGEKLAAELAGSLTQSEYENRRLIRDAIDLYYRDQIARTEL  3329562

Query  121      DAQRFTLMRMASQADLMIDLHCDWDALPHLYTTPHAWQDIEPLARWLGSEVQLLAQISGG  180
                ++QR+TLMRMASQADLMIDLHCDWDALPHLYTTPHAW ++EPLARWLGSEVQLLAQISGG
Sbjct  3329563  ESQRYTLMRMASQADLMIDLHCDWDALPHLYTTPHAWSEVEPLARWLGSEVQLLAQISGG  3329742

Query  181      EPFDEACCEPWLTLAERFGGEYPMPRGLLPVTLELRGVADVSPGQAEKDADAIINALIEG  240
                EPFDEACCEPWLTLA+RFG +YPMPRGLLPVTLELRGVADVS  QAEKDADAIINAL E
Sbjct  3329743  EPFDEACCEPWLTLAKRFGDDYPMPRGLLPVTLELRGVADVSAEQAEKDADAIINALTES  3329922

Query  241      GYIAGDVGESPALINPATPLAGCEYIHAPHSGMLLNRRELGEWIKPGEVVAEIVDPITDQ  300
                GYI+GD G SPAL +P TPLAGCEYIHAP+SG+LLNRR+LGEWIKPGEVVAE++DP++D
Sbjct  3329923  GYISGDPGVSPALRHPPTPLAGCEYIHAPYSGLLLNRRKLGEWIKPGEVVAEVIDPVSDH  3330102

Query  301      VTPLVAEFGGVLYARNLMKFATAGMLVVK  329
                +TPLVAE+GGVLYARNL KF T+GML V+
Sbjct  3330103  ITPLVAEYGGVLYARNLTKFVTSGMLAVR  3330189
```

Plautia stali symbiont DNA, complete genome
Sequence ID: **AP012551.1** Length: 4035456 Number of Matches: 2
Range 1: 3907231 to 3908091

Score	Expect	Method	Identities	Positives	Gaps	Frame
535 bits(1377)	5e-170()	Compositional matrix adjust.	257/287(90%)	273/287(95%)	0/287(0%)	+1

Features:

```
Query  1        SQQRVYIQAALHGDELPGMAVAWYLKHKLLALESAGQLKSKITLVPVANPLAMGQHWHGS  60
                SQQRVYIQAALHGDELPGMAVAWYLK KLLALESAG+LK+KITLVPVANPLA+GQHWHG+
Sbjct  3907231  SQQRVYIQAALHGDELPGMAVAWYLKQKLLALESAGKLKAKITLVPVANPLALGQHWHGT  3907410

Query  61       HLGRFHTLSGQDFNRRFPALGDTLAEELAGSLTQSEYENKRLIRDAIDRHYRDRVARTEL  120
                HLGRFHTLSGQDF RRFPALGDTLA ELAGSLTQSEYENKRLIRDAIDRHYRDR+A++EL
Sbjct  3907411  HLGRFHTLSGQDFYRRFPALGDTLAAELAGSLTQSEYENKRLIRDAIDRHYRDRIAKSEL  3907590

Query  121      DAQRFTLMRMASQADLMIDLHCDWDALPHLYTTPHAWQDIEPLARWLGSEVQLLAQISGG  180
                +AQRFTLMR+AS ADLMIDLHCDWDALPHLYTTPHAW +IEPLARWLGSEVQLLAQISGG
Sbjct  3907591  EAQRFTLMRIAS*ADLMIDLHCDWDALPHLYTTPHAWSEIEPLARWLGSEVQLLAQISGG  3907770

Query  181      EPFDEACCEPWLTLAERFGGEYPMPRGLLPVTLELRGVADVSPGQAEKDADAIINALIEG  240
                EPFDEACCEPWLTLA RFG +YPMPRGLLPVTLELRGVADVSP QAEKDADAIINALIEG
Sbjct  3907771  EPFDEACCEPWLTLAGRFGKDYPMPRGLLPVTLELRGVADVSPEQAEKDADAIINALIEG  3907950

Query  241      GYIAGDVGESPALINPATPLAGCEYIHAPHSGMLLNRRELGEWIKPG  287
                GYIAG+ G+SPALI+  T LAGCEYIHAPHSG+LLNRR+LGEWI  G
Sbjct  3907951  GYIAGETGDSPALIHAPTQLAGCEYIHAPHSGLLLNRRKLGEWISGG  3908091
```

Range 2: 3908078 to 3908206

Score	Expect	Method	Identities	Positives	Gaps	Frame
77.4 bits(189)	1e-11()	Compositional matrix adjust.	37/43(86%)	38/43(88%)	0/43(0%)	+2

Features:

```
Query  287      GEVVAEIVDPITDQVTPLVAEFGGVLYARNLMKFATAGMLVVK  329
                G VVAEIVDPI D VTPLVAEFGGVLYARNL KF TAGMLVV+
Sbjct  3908078  GLVVAEIVDPINDYVTPLVAEFGGVLYARNLTKFVTAGMLVVR  3908206
```

BLAST is a registered trademark of the National Library of Medicine

Support center Mailing list YouTube

- National Library Of Medicine
- National Institutes Of Health
- U.S. Department of Health & Human Services
- USA.gov

NCBI
National Center for Biotechnology Information, U.S. National Library of Medicine 8600 Rockville Pike, Bethesda MD, 20894 USA
Policies and Guidelines | Contact
