# Supplementary material for: Grafting or pruning in the animal tree: lateral gene transfer and gene loss?
Source: BMC Genomics. 2018 Jun 18;19:470. doi: 10.1186/s12864-018-4832-5 (PMC6006793; doi:10.1186/s12864-018-4832-5)
Supplement: Supplementary file 3 — BLASTN search of A. pisum strain LSR1 unplaced genomic scaffold, Acyr_2.0 Scaffold2139 (NW_003385628.1) against NT allowing for 20,000 matches with an e-value below 0.00001. (PDF 284 kb) [file 12864_2018_4832_MOESM3_ESM.pdf]

BLAST ® » blastn suite » RID-9JT540KY014

BLAST Results

Questions/comments

Job title: ref|NW_003385628.1| (7613 letters)

**RID**	9JT540KY014 (Expires on 03-03 21:50 pm)		
**Query ID**	NW_003385628.1	**Database Name**	nr
**Description**	Acyrthosiphon pisum strain LSR1 unplaced genomic scaffold, Acyr_2.0 Scaffold2139	**Description**	Nucleotide collection (nt)
**Molecule type**	dna	**Program**	BLASTN 2.8.0+
**Query Length**	7613		

Graphic Summary

Distribution of the top 102 Blast Hits on 106 subject sequences

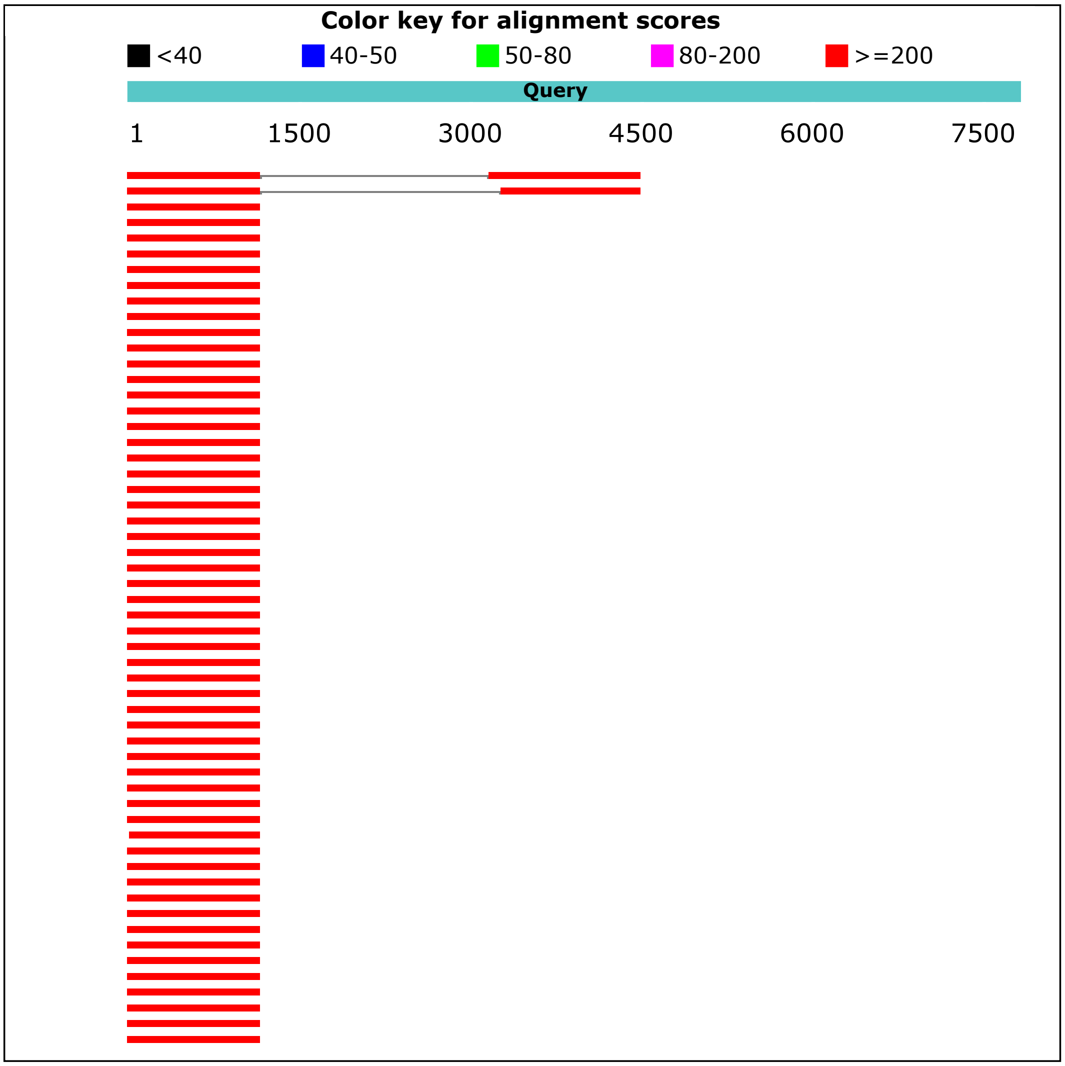

Descriptions

Sequences producing significant alignments:

Description	Max score	Total score	Query cover	E value	Ident	Accession
Pantoea rwandensis strain ND04, complete genome	1855	3702	31%	0.0	92%	CP009454.1
Pantoea vagans strain ND02, complete genome	1471	2569	30%	0.0	90%	CP011427.1
Plautia stali symbiont DNA, complete genome	1203	1203	14%	0.0	86%	AP012551.1
Pantoea sp. At-9b, complete genome	1149	1149	14%	0.0	85%	CP002433.1
Pantoea sp. PSNIH1, complete genome	1077	1077	14%	0.0	84%	CP009880.2
Pantoea ananatis LMG 20103, complete genome	1050	1050	14%	0.0	84%	CP001875.2
Pantoea ananatis AJ13355 DNA, complete genome	1038	1038	14%	0.0	83%	AP012032.2
Pantoea ananatis strain R100, complete genome	1038	1038	14%	0.0	83%	CP014207.1
Pantoea ananatis LMG 5342 main chromosome complete genome	1038	1038	14%	0.0	83%	HE617160.1
Pantoea ananatis strain 97-1 genome	1027	1027	14%	0.0	83%	CP020943.1
Pantoea ananatis strain YJ76, complete genome	1022	1022	14%	0.0	83%	CP022427.1
Pantoea ananatis PA13, complete genome	1022	1022	14%	0.0	83%	CP003085.1
Pantoea stewartii subsp. stewartii DC283, complete genome	998	998	14%	0.0	83%	CP017581.1
Erwinia sp. EM595 genome assembly EM595, chromosome : 1	905	905	14%	0.0	81%	LN907827.1
Erwinia billingiae strain Eb661 complete chromosome	828	828	14%	0.0	80%	FP236843.1
Serratia marcescens strain SOLR4 chromosome	754	754	14%	0.0	79%	CP025698.1
Serratia sp. FS14, complete genome	750	750	14%	0.0	79%	CP005927.1
Serratia marcescens strain UMH2, complete genome	749	749	14%	0.0	79%	CP018924.1
Serratia marcescens strain UMH7, complete genome	749	749	14%	0.0	79%	CP018919.1
Serratia marcescens strain RSC-14, complete genome	749	749	14%	0.0	79%	CP012639.1
Gibbsiella quercinecans strain FRB97, complete genome	745	745	14%	0.0	79%	CP014136.1
Serratia marcescens strain S2I7 genome	745	745	14%	0.0	79%	CP021984.1
Serratia marcescens WW4, complete genome	745	745	14%	0.0	79%	CP003959.1
Serratia sp. JKS000199 genome assembly, chromosome: I	743	743	14%	0.0	79%	LT907843.1
Cronobacter turicensis z3032 complete genome	739	739	14%	0.0	79%	FN543093.2
Serratia marcescens strain UMH3, complete genome	737	737	14%	0.0	79%	CP018925.1
Serratia proteamaculans 568, complete genome	734	734	14%	0.0	79%	CP000826.1

Description	Max score	Total score	Query cover	E value	Ident	Accession
Enterobacter cloacae subsp. cloacae ATCC 13047, complete genome	728	728	14%	0.0	79%	CP001918.1
Serratia liquefaciens ATCC 27592, complete genome	723	723	14%	0.0	79%	CP006252.1
Serratia liquefaciens strain FDAARGOS_125 chromosome, complete genome	717	717	14%	0.0	78%	CP014017.2
Escherichia coli strain AR_0019 chromosome, complete genome	717	717	14%	0.0	78%	CP024889.1
Escherichia coli isolate Co6114 genome	717	717	14%	0.0	78%	CP016034.1
Serratia liquefaciens strain HUMV-21, complete genome	717	717	14%	0.0	78%	CP011303.1
Enterobacter hormaechei subsp. hormaechei strain 34983, complete genome	713	713	14%	0.0	78%	CP010377.1
Enterobacter cloacae strain M12X01451 chromosome complete genome	712	712	14%	0.0	78%	CP017475.1
Kluyvera georgiana strain YDC799, complete genome	708	708	14%	0.0	78%	CP022114.1
Enterobacter cloacae subsp. dissolvens SDM, complete genome	704	704	14%	0.0	78%	CP003678.1
Enterobacter cloacae isolate SBP-8 genome	701	701	14%	0.0	78%	CP016906.1
Enterobacter cloacae strain GGT036, complete genome	701	701	14%	0.0	78%	CP009756.1
Serratia rubidaea strain 1122, complete genome	695	695	14%	0.0	78%	CP014474.1
Enterobacter sp. DKU_NT_01 chromosome	693	693	14%	0.0	78%	CP021137.1
Enterobacter cloacae strain DG6 genome assembly, chromosome: I	693	693	14%	0.0	78%	LT840187.1
Enterobacter cloacae strain FRM, complete genome	689	689	14%	0.0	78%	CP019889.1
Enterobacter cloacae strain AR_0072, complete genome	688	688	14%	0.0	78%	CP026850.1
Enterobacter hormaechei subsp. oharae strain DSM 16687, complete genome	688	688	14%	0.0	78%	CP017180.1
Lelliottia sp. PFL01, complete genome	684	684	14%	0.0	78%	CP018628.1
Enterobacter cloacae strain AR_0163, complete genome	684	684	14%	0.0	78%	CP021749.1
Klebsiella quasipneumoniae strain HKUOPL4, complete genome	678	678	14%	0.0	78%	CP014156.1
Klebsiella quasipneumoniae strain HKUOPJ4, complete genome	678	678	14%	0.0	78%	CP014155.1
Klebsiella quasipneumoniae strain HKUOPA4, complete genome	678	678	14%	0.0	78%	CP014154.1
Klebsiella pneumoniae subsp. pneumoniae strain HKUOPLC, complete genome	678	678	14%	0.0	78%	CP012300.1
Klebsiella variicola strain HKUOPLA, complete genome	678	678	14%	0.0	78%	CP012252.1
Citrobacter werkmanii strain BF-6, complete genome	673	673	14%	0.0	78%	CP019986.1
Enterobacter cloacae subsp. cloacae DNA, nearly complete genome, strain: 2016_smu953	669	669	14%	0.0	78%	AP018340.1
Klebsiella quasivariicola strain KPN1705 chromosome, complete genome	667	667	14%	0.0	78%	CP022823.1

Description	Max score	Total score	Query cover	E value	Ident	Accession
Enterobacter cloacae strain MS7884A, complete genome	665	665	14%	0.0	78%	CP022532.1
Enterobacter hormaechei subsp. steigerwaltii strain DSM 16691, complete genome	662	662	14%	0.0	77%	CP017179.1
Citrobacter freundii CFNIH1, complete genome	662	662	14%	0.0	78%	CP007557.1
Enterobacter cloacae strain AR_0060 chromosome, complete genome	660	660	14%	0.0	77%	CP026719.1
Raoultella ornithinolytica strain S12, complete genome	660	660	14%	0.0	78%	CP010557.1
Klebsiella quasipneumoniae strain ATCC 700603 chromosome, complete genome	656	656	14%	0.0	78%	CP014071.1
Klebsiella quasipneumoniae strain ATCC 700603, complete genome	656	656	14%	0.0	78%	CP014696.2
Klebsiella pneumoniae strain KSB1_10J chromosome, complete genome	651	651	14%	0.0	78%	CP024515.1
Klebsiella pneumoniae strain KSB2_1B chromosome, complete genome	651	651	14%	0.0	78%	CP024504.1
Klebsiella pneumoniae strain KSB1_4E chromosome, complete genome	651	651	14%	0.0	78%	CP024499.1
Klebsiella pneumoniae strain KSB1_7E chromosome, complete genome	651	651	14%	0.0	78%	CP024496.1
Enterobacter cloacae strain AR_0136, complete genome	651	651	14%	0.0	77%	CP021902.1
Enterobacter cloacae strain AR_0053, complete genome	651	651	14%	0.0	77%	CP021776.1
Enterobacter cloacae strain AR_0002, complete genome	651	651	14%	0.0	77%	CP018814.1
Enterobacter hormaechei subsp. oharae strain 34978, complete genome	651	651	14%	0.0	77%	CP012165.1
Leclercia sp. LSNIH3 chromosome, complete genome	645	645	14%	6e-180	77%	CP026387.1
Dickeya paradisiaca Ech703 chromosome, complete genome	645	645	14%	6e-180	77%	CP001654.1
Lelliottia amnigena strain FDAARGOS_395 chromosome, complete genome	640	640	14%	3e-178	77%	CP023529.1
Enterobacter xiangfangensis strain LMG27195, complete genome	640	640	14%	3e-178	77%	CP017183.1
Leclercia adecarboxylata strain USDA-ARS-USMARC-60222, complete genome	640	640	14%	3e-178	77%	CP013990.1
Pectobacterium carotovorum strain SCC1 chromosome	623	623	14%	3e-173	77%	CP021894.1
Kosakonia sacchari strain BO-1, complete genome	614	614	14%	2e-170	77%	CP016337.1
Pectobacterium polaris strain NIBIO1392 chromosome, complete genome	606	606	14%	3e-168	77%	CP017482.1
Enterobacteriaceae bacterium strain FGI 57, complete genome	603	603	14%	4e-167	77%	CP003938.1
Lelliottia amnigena strain ZB04, complete genome	601	601	14%	1e-166	76%	CP015774.2
Pectobacterium polaris strain NIBIO1006 chromosome, complete genome	584	584	14%	1e-161	76%	CP017481.1
Klebsiella oxytoca strain CAV1015, complete genome	584	584	14%	1e-161	77%	CP017928.1

Description	Max score	Total score	Query cover	E value	Ident	Accession
Klebsiella oxytoca strain CAV1335, complete genome	584	584	14%	1e-161	77%	CP011618.1
Klebsiella oxytoca strain CAV1099, complete genome	584	584	14%	1e-161	77%	CP011597.1
Pectobacterium carotovorum subsp. odoriferum strain BC S7, complete genome	569	569	14%	4e-157	76%	CP009678.1
Enterobacter cancerogenus strain CR-Eb1 chromosome, complete genome	560	560	14%	2e-154	76%	CP025225.1
Pectobacterium atrosepticum strain 36A chromosome, complete genome	518	518	14%	1e-141	75%	CP024956.1
Pectobacterium atrosepticum strain 21A, complete genome	518	518	14%	1e-141	75%	CP009125.1
Pectobacterium atrosepticum strain JG10-08, complete genome	518	518	14%	1e-141	75%	CP007744.1
Rouxiella sp. ERMR1:05 chromosome, complete genome	492	492	8%	8e-134	80%	CP019062.1
Rahnella aquatilis CIP 78.65 = ATCC 33071, complete genome	459	459	8%	8e-124	79%	CP003244.1
Enterobacteriaceae bacterium bta3-1, complete genome	442	442	14%	8e-119	74%	CP004083.1
Rahnella aquatilis HX2, complete genome	420	420	8%	4e-112	78%	CP003403.1
Edwardsiella hoshinae strain ATCC 35051, complete genome	379	379	14%	6e-100	73%	CP016043.1
Vibrio fluvialis strain 12605 chromosome 1, complete sequence	351	351	8%	1e-91	77%	CP019118.1
Tolumonas auensis DSM 9187, complete genome	342	342	6%	9e-89	80%	CP001616.1
Vibrio coralliilyticus strain 58 chromosome I, complete sequence	302	302	8%	1e-76	75%	CP016556.1
Aeromonas veronii strain CB51, complete genome	298	298	7%	2e-75	76%	CP015448.1
Vibrio coralliilyticus strain RE98 chromosome 1, complete sequence	291	291	8%	3e-73	75%	CP009617.1
Vibrio coralliilyticus strain OCN014 chromosome 1, complete sequence	279	279	8%	7e-70	75%	CP009264.2
Vibrio vulnificus strain CECT 4999 chromosome I, complete sequence	276	276	8%	9e-69	75%	CP014636.1
Vibrio vulnificus strain FORC_037 chromosome I, complete sequence	270	270	8%	4e-67	75%	CP016321.1
Klebsiella quasipneumoniae subsp. similipneumoniae DNA, lipopolysaccharide biosynthesis gene cluster, complete sequence, serotype: O12:K80, strain: 708	267	267	5%	5e-66	78%	AB795942.1
Vibrio vulnificus strain FORC_054 chromosome 1, complete sequence	265	265	8%	2e-65	74%	CP019121.1
Vibrio vulnificus NBRC 15645 = ATCC 27562 chromosome 1, complete sequence	265	265	8%	2e-65	74%	CP012881.1
Klebsiella pneumoniae DNA, lipopolysaccharide biosynthesis gene cluster, serotype: O8, complete sequence, strain: 889	244	244	5%	2e-59	78%	AB819963.1

Alignments

Pantoea rwandensis strain ND04, complete genome
Sequence ID: **CP009454.1** Length: 4327607 Number of Matches: 2
Range 1: 2084962 to 2086262

Score	Expect	Identities	Gaps	Strand	Frame
1855 bits(1004)	0.0()	1203/1302(92%)	2/1302(0%)	Plus/Minus	

Features:

```
Query  3087     CAGAAGAAGTACTTCGCCACCGACATCTCGATCCAGCAGTAATCAATGTAGAGGCGGTCT  3146
                |||||||||||||||||||| |||||||||||||||||| |||||||| || ||||||||
Sbjct  2086262  CAGAAGAAGTACTTCGCCACTGACATCTCGATCCAGCATTAATCAATGCAGGGGCGGTCT  2086203

Query  3147     CAAGGCCGCCCTATTCGCGCCGCTAACATGAGCGAGCGCAAATTTAGCGGCTTCATTTCC  3206
                  |||||||||| ||||||| ||||||||||||||||||||||||||||||||||  ||
Sbjct  2086202  TCAGGCCGCCCTGTTCGCGCTGCTAACATGAGCGAGCGCAAATTTAGCGGCTTCATCCCC  2086143

Query  3207     GCTCATTCTCACAGCAGCACTATAATTCCAATCAATAAAATTAATCAGGCACGACGTTCA  3266
                ||||||| |||||||||||||||||||||| |||||||||||||||||||||||||||||
Sbjct  2086142  GCTCATTTTCACAGCAGCACTATAATTCCAGTCAATAAAATTAATCAGGCACGACGTTCA  2086083

Query  3267     ACGTGCCGTAGATACAGGACATCGACCTCGTCATGCAACAACAACATCATCCTC-TGCTA  3325
                  |||||||||||| ||||||||||||| || ||||||||||||||||||||| || |||||
Sbjct  2086082  GTGTGCCGTAGATATAGGACATCGACCGCGCCATGCAACAACAACATCATC-TCTTGCTA  2086024

Query  3326     AGTGCTTCCCTCGGTACACAGCGCGAAATCGTCAGCTTCCATTTCGGTACCGATTCCCAG  3385
                |||||||||||||||||||||||||||||||||||||| |||||||||||||||||||||
Sbjct  2086023  AGTGCTTCCCTCGGTACACAGCGCGAAATCGTCAGCTTTCATTTCGGTACCGATTCCCAG  2085964

Query  3386     CAGCGCGTCTACATTCAGGCGGCACTGCACGGTGATGAACTGCCGGGTATGGCGGTGGCG  3445
                || |||||||||||||||||||||||||||||||||||||||||||||||||||||||||
Sbjct  2085963  CAACGCGTCTACATTCAGGCGGCACTGCACGGTGATGAACTGCCGGGTATGGCGGTGGCG  2085904

Query  3446     TGGTATCTGAAACACAAGCTGCTGGCGCTGGAATCGGCCGGGCAGCTCAAATCCAAAATT  3505
                ||||||||||||||||||||||||||||||||||| ||||||||||||||||||||||||
Sbjct  2085903  TGGTATCTGAAACACAAGCTGCTGGCGCTGGAATCTGCCGGGCAGCTCAAATCCAAAATT  2085844

Query  3506     ACGCTGGTGCCGGTGGCAAATCCGCTGGCGATGGGGCAACACTGGCACGGCAGCCATTTG  3565
                ||||||||||||||||| |||||||||||||||||| ||||||||||||||||| ||||||
Sbjct  2085843  ACGCTGGTGCCGGTGGCCAATCCGCTGGCGATGGGTCAACACTGGCACGGCAGTCATTTG  2085784

Query  3566     GGTCGATTCCATACGCTTTCAGGTCAGGATTTCAATCGCCGTTTCCCGGCATTAGGCGAT  3625
                |||||||||||||||||||||||||||||||||||||||||||||||||| |||||||||
Sbjct  2085783  GGTCGATTCCATACGCTTTCAGGTCAGGATTTCAATCGCCGTTTCCCGGCGTTAGGCGAT  2085724

Query  3626     ACCCTGGCTGAAGAGTTGGCCGGTTCGTTGACACAGAGTGAATATGAGAACAAACGGTTG  3685
                |||||||||||||||||||||||||||||||| || || ||||||||||||||||| ||
Sbjct  2085723  ACCCTGGCTGAAGAGTTGGCCGGTTCGTTGACGCAAAGCGAATATGAGAACAAACGGCTG  2085664

Query  3686     ATCCGTGATGCCATTGATCGCCACTATCGAGACCGCGTTGCTCGTACCGAACTGGATGCG  3745
                ||||||||||||||||||||||||||||| || ||| ||||||||||||||||||||||||
Sbjct  2085663  ATCCGTGATGCCATTGATCGCCACTATCGCGATCGCATTGCTCGTACCGAACTGGATGCG  2085604

Query  3746     CAGCGCTTCACGCTAATGCGTATGGCCAGCCAGGCAGATTTGATGATTGATCTGCACTGC  3805
                ||||||||||||||||||||||||||||||||||||||||||||||||||||||||||||
Sbjct  2085603  CAGCGCTTCACGCTAATGCGTATGGCCAGCCAGGCAGATTTGATGATTGATCTGCACTGC  2085544

Query  3806     GACTGGGACGCATTGCCGCATCTCTACACGACCCCGCATGCGTGGCAGGATATCGAGCCT  3865
                ||||||||||||||||||||||| || || || ||||| || ||||||||||||||| ||
Sbjct  2085543  GACTGGGACGCATTGCCGCATCTGTATACCACGCCGCACGCATGGCAGGATATCGAACCA  2085484

Query  3866     TTAGCGCGTTGGTTAGGTAGCGAAGTACAGCTTCTGGCCCAAATCTCTGGTGGCGAGCCG  3925
                ||||||||||||||||| || ||||| || || ||||| |||||| || ||||||||| ||
Sbjct  2085483  TTAGCGCGTTGGTTAGGCAGTGAAGTGCAACTGCTGGCGCAAATATCCGGTGGCGAACCC  2085424

Query  3926     TTTGATGAAGCCTGCTGCGAGCCGTGGTTAACGTTGGCCGAGCGGTTTGGTGGTGAGTAT  3985
                |||||||| ||||||||| || |||||| | ||  |||| ||||| ||||||||||||||||
Sbjct  2085423  TTTGATGAGGCCTGCTGTGAACCGTGGCTGACACTGGCTGAGCGATTTGGTGGTGAGTAT  2085364

Query  3986     CCGATGCCACGGGGTTTGTTACCGGTGACGCTCGAATTACGTGGTGTGGCGGATGTGTCG  4045
                |||||||| || |||||||| || |||||||| || |||||||| || |||||||||||||
Sbjct  2085363  CCGATGCCTCGTGGTTTGTTGCCAGTGACGCTGGAGTTACGTGGGGTTGCGGATGTGTCG  2085304

Query  4046     CCTGGACAAGCTGAGAAGGATGCGGATGCGATTATTAACGCGCTGATTGAAGGTGGCTAC  4105
                ||   ||| || ||||||||||||||||||||||||||||| |||||||||| ||||| 
Sbjct  2085303  CCGCAACAGGCGGAGAAGGATGCGGATGCGATTATTAACGCGTTGATTGAAGGCGGCTAT  2085244

Query  4106     ATTGCGGGTGACGTGGGTGAGTCCCCTGCTCTGATCAATCCTGCGACACCGCTGGCAGGT  4165
                || ||||||||    |||||||||||||| ||||||||||| ||||||||||||||||||
Sbjct  2085243  ATCGCGGGTGAAACAGGTGAGTCCCCTGCCCTGATCAATCCCGCGACACCGCTGGCAGGT  2085184

Query  4166     TGTGAGTATATCCATGCACCGCATTCTGGAATGCTGTTGAATCGCCGTGAGTTGGGTGAG  4225
                |||||||||||||||||||||||||||||| |||||||||||||||||||||||||||||
Sbjct  2085183  TGTGAGTATATCCATGCACCGCATTCTGGATTGCTGTTGAATCGCCGTGAGTTGGGTGAG  2085124

Query  4226     TGGATCAAGCCGGGTGAAGTGGTAGCGGAGATTGTGGACCCGATTACCGACCAGGTGACG  4285
                ||||||||||||||||||||||| |||||||||||||| ||||||||||||||||||| 
Sbjct  2085123  TGGATCAAGCCGGGTGAAGTGGTGGCGGAGATTGTGGATCCGATTACCGACCAGGTGACA  2085064

Query  4286     CCGCTGGTGGCAGAGTTTGGTGGGGTGCTGTATGCAAGGAACCTGATGAAGTTTGCTACG  4345
                |||||||||||||||||||||||||||||||||||  | || || ||||||||||||||||
Sbjct  2085063  CCGCTGGTGGCAGAGTTTGGTGGGGTGCTGTATGCGCGAAATCTCATGAAGTTTGCTACG  2085004

Query  4346     GCTGGGATGCTGGTGGTGCGGTTGGCGGGTGAAAATGCGGGC  4387
                || || |||||||||||  || | ||||| ||||||||||||
Sbjct  2085003  GCGGGAATGCTGGTGGTAAGGCTTGCGGGGGAAAATGCGGGC  2084962
```

Range 2: 2087356 to 2088478

Score	Expect	Identities	Gaps	Strand	Frame
1847 bits(1000)	0.0()	1082/1123(96%)	0/1123(0%)	Plus/Minus	

Features:

```
Query  1        TCGTGCCACTGGCGCAGCGCTATGCGCAGGAAGGCGCTGACGAGCTGGTGTTCTATGATA  60
Sbjct  2088478  TCGTGCCACTGGCGCAGCGCTATGCGCAGGAAGGCGCTGACGAGCTGGTGTTCTATGATA  2088419
Query  61       TCACCGCCTCTTCCGATGGCCGTGTGGTGGATAAAAGCTGGGTGTCACGCGTCGCAGAAG  120
Sbjct  2088418  TCACCGCCTCTTCCGATGGCCGCGTGGTGGATAAAAGCTGGGTGTCACGCGTCGCAGAAG  2088359
Query  121      TGATTGATATTCCGTTCTGCGTGGCGGGCGGTATCAAGAGTGAGGAGGATGCCGCGCGCA  180
Sbjct  2088358  TGATTGATATTCCGTTCTGCGTGGCGGGCGGTATCAAGAGTGAGGAGGATGCCGCGCGCA  2088299
Query  181      TTCTGCAGTTCGGTGCGGATAAGATCTCCATCAACTCCCCGGCGCTGGCTGACCCTACGC  240
Sbjct  2088298  TTCTGCAGTTCGGTGCGGATAAGATCTCCATCAACTCCCCGGCGCTGGCTGACCCGACGC  2088239
Query  241      TGATCACGCGTCTGGCAGATCGCTTTGGTGTGCAGTGTATCGTGGTGGGTATTGATACCT  300
Sbjct  2088238  TGATCACGCGTCTGGCGGATCGCTTTGGTGTGCAGTGCATCGTGGTGGGTATTGATACCT  2088179
Query  301      GGTTCGATGAAGCCACCGGCAAGTATCACGTCAATCAATATACCGGCGATGAATCACGTA  360
Sbjct  2088178  GGTTTGATGAAGCCACAGGCAAGTATCACGTCAACCAGTATACCGGCGATGAGTCACGTA  2088119
Query  361      CCCGTGTCACCACCTGGGAAACCCTGGATTGGGTACAGGAAGTGCAGAAGCTAGGCGCGG  420
Sbjct  2088118  CCCGTGTCACCACCTGGGAAACCCTGGATTGGGTGCAGGAAGTGCAGAAGCTGGGTGCGG  2088059
Query  421      GCGAAATCGTGCTGAACATGATGAACCAGGATGGCGTACGTAATGGCTACGATCTGGTGC  480
Sbjct  2088058  GCGAAATCGTGCTGAACATGATGAACCAGGATGGCGTACGTAATGGCTACGATCTGGTGC  2087999
Query  481      AGCTGAAGAAAGTGCGTGAGGTATGTAAAGTGCCCCTCATCGCTTCTGGCGGCGCGGGCA  540
Sbjct  2087998  AGCTGAAGAAAGTCCGAGACGTGTGTAAAGTGCCCCTCATCGCTTCTGGCGGAGCAGGCA  2087939
Query  541      CCATGCCACACTTTCTCGAAGCCTTTGAACAGGCAAACGTTGATGGCGCACTGGCCGCTT  600
Sbjct  2087938  CCATGCCGCATTTCCTCGAAGCCTTTGAACAGGCAAACGTCGATGGTGCGCTGGCCGCTT  2087879
Query  601      CGGTGTTTCACAAACAAATTATCAATATCGGCGAGTTGAAAAACTTTCTGATCGACAACG  660
Sbjct  2087878  CAGTGTTTCACAAACAAATTATTAATATCGGCGAGTTGAAAAACTTCCTGATCGACAACG  2087819
Query  661      GTGTGGAGATTCGCGCGTGTTAACTGCAGAACAACTGGCCAAACTGGATTGGGCCAAAAC  720
Sbjct  2087818  GTGTGGAGATTCGCGCGTGTTAACTGCAGAACAACTGGCCAAACTGGATTGGGCCAAAAC  2087759
Query  721      CGCGGGCATGATGCCCGTCATCGTCCAGCACAACGTTTCTGGCGAAGTGCTGATGCACGG  780
Sbjct  2087758  CGCGGGCATGATGCCCGTCATCGTCCAGCACAACGTTTCTGGCGAAGTGCTGATGCACGG  2087699
Query  781      TTATATGAATGAGGAAGCGCTGCAAAAGACGCTGGCGGAAGGCAATGTCACTTTCTTCTC  840
Sbjct  2087698  TTATATGAATGAGGAAGCACTGCAAAAGACGCTGGCGGAAGGCAACGTCACTTTCTTCTC  2087639
Query  841      TCGCACCAAAAACCGCTTGTGGACCAAGGGGGAAACCTCCGGTCACTTTTTGAAGGTCGC  900
Sbjct  2087638  TCGCACCAAAAACCGCTTGTGGACCAAAGGGGAAACCTCCGGTCACTTCCTGCAGGTCGC  2087579
Query  901      GAGCATCACGCCGGATTGTGATAACGACACCCTGCTAGTGCTGGCAAACCCGATTGGGCC  960
Sbjct  2087578  GAGCATCACGCCGGATTGTGATAACGACACCCTGCTGGTGCTGGCAAACCCGATTGGGCC  2087519
Query  961      AACCTGCCACCTTGGCACCTCAAGCTGCTTCTCACCGGCTGCGCCAGATTGGGCGTTCCT  1020
Sbjct  2087518  AACCTGCCACCTCGGAACATCAAGCTGCTTCTCTCCAGCTGCGCCAGATTGGGCGTTCCT  2087459
Query  1021     GTATCAGCTGGAGCAGCTGCTGGCTTCACGTAAGAGCGCCGATCCTGAGAGTTCTTACAC  1080
Sbjct  2087458  GTATCAGCTGGAGCAGCTGCTGGCTTCACGTAAGAGCGCCGATCCTGAGAGTTCTTACAC  2087399
Query  1081     CGCGAAACTCTATGCCAGCGGCACAAAGCGTATCGCGCAGAAA  1123
Sbjct  2087398  CGCGAAACTGTATGCCAGCGGCACCAAGCGTATCGCGCAGAAA  2087356
```

Pantoea vagans strain ND02, complete genome

Sequence ID: **CP011427.1** Length: 4313264 Number of Matches: 2

Range 1: 1341479 to 1342601

Score	Expect	Identities	Gaps	Strand	Frame
1471 bits(796)	0.0()	1014/1123(90%)	0/1123(0%)	Plus/Minus	

Features:

```
Query  1        TCGTGCCACTGGCGCAGCGCTATGCGCAGGAAGGCGCTGACGAGCTGGTGTTCTATGATA  60
Sbjct  1342601  TCGTGCCGCTGGCGCAGCGTTATGCGCAGGAAGGCGCTGACGAGCTGGTGTTCTATGATA  1342542

Query  61       TCACCGCCTCTTCCGATGGCCGTGTGGTGGATAAAAGCTGGGTGTCACGCGTCGCAGAAG  120
Sbjct  1342541  TCACCGCCTCTTCAGATGGCCGTGTCGTCGATAAAAGCTGGGTGTCACGCGTTGCGGAAG  1342482

Query  121      TGATTGATATTCCGTTCTGCGTGGCGGGCGGTATCAAGAGTGAGGAGGATGCCGCGCGCA  180
Sbjct  1342481  TGATCGATATTCCTTTCTGCGTGGCCGGTGGCATTAAAACCCCGGAAGACGCAGCGCGCA  1342422

Query  181      TTCTGCAGTTCGGTGCGGATAAGATCTCCATCAACTCCCCGGCGCTGGCTGACCCTACGC  240
Sbjct  1342421  TTCTGCAGTTCGGTGCGGACAAGATCTCCATCAACTCCCCCGCACTGGCAGACCCGACGC  1342362

Query  241      TGATCACGCGTCTGGCAGATCGCTTTGGTGTGCAGTGTATCGTGGTGGGTATTGATACCT  300
Sbjct  1342361  TGATTACCCGTCTGGCCGATCGCTTTGGCGTGCAGTGTATCGTGGTAGGCATTGATACCT  1342302

Query  301      GGTTCGATGAAGCCACCGGCAAGTATCACGTCAATCAATATACCGGCGATGAATCACGTA  360
Sbjct  1342301  GGTTCGATGAAGCAACCGGCAAGTATCACGTTAACCAGTACACCGGTGACGAATCACGTA  1342242

Query  361      CCCGTGTCACCACCTGGGAAACCCTGGATTGGGTACAGGAAGTGCAGAAGCTAGGCGCGG  420
Sbjct  1342241  CCCGTGTCACCACCTGGGAAACCCTCGACTGGGTGCAGGAAGTGCAGAAGTTGGGCGCGG  1342182

Query  421      GCGAAATCGTGCTGAACATGATGAACCAGGATGGCGTACGTAATGGCTACGATCTGGTGC  480
Sbjct  1342181  GCGAAATCGTACTGAATATGATGAACCAGGATGGCGTGCGCAACGGCTATGATCTGATAC  1342122

Query  481      AGCTGAAGAAAGTGCGTGAGGTATGTAAAGTGCCCCTCATCGCTTCTGGCGGCGCGGGCA  540
Sbjct  1342121  AGCTGAAGAAAGTGCGTGACGTGTGTAGAGTGCCACTGATCGCCTCTGGCGGCGCGGGCA  1342062

Query  541      CCATGCCACACTTTCTCGAAGCCTTTGAACAGGCAAACGTTGATGGCGCACTGGCCGCTT  600
Sbjct  1342061  CCATGCCGCACTTCCTCGAAGCCTTCGAGCAAGCGAACGTGGATGGCGCTCTGGCTGCTT  1342002

Query  601      CGGTGTTTCACAAACAAATTATCAATATCGGCGAGTTGAAAAACTTTCTGATCGACAACG  660
Sbjct  1342001  CCGTGTTTCACAAACAAATTATCAATATCGGCGAGTTGAAAAACTTCCTGATCGACAACG  1341942

Query  661      GTGTGGAGATTCGCGCGTGTTAACTGCAGAACAACTGGCCAAACTGGATTGGGCCAAAAC  720
Sbjct  1341941  GTGTGGAGATTCGCGCGTGTTAACTGCAGAACAACTGGCCAAACTGGATTGGGCCAAAAC  1341882

Query  721      CGCGGGCATGATGCCCGTCATCGTCCAGCACAACGTTTCTGGCGAAGTGCTGATGCACGG  780
Sbjct  1341881  CGCGGGCATGATGCCTGTTATCGTCCAGCACAATGTCTCTGGCGAAGTGCTGATGCACGG  1341822

Query  781      TTATATGAATGAGGAAGCGCTGCAAAAGACGCTGGCGGAAGGCAATGTCACTTTCTTCTC  840
Sbjct  1341821  TTATATGAATGAGGAAGCGCTGCAAAAGACGCTGGCGGAAGGCAACGTCACCTTCTTCTC  1341762

Query  841      TCGCACCAAAAACCGCTTGTGGACCAAGGGGGAAACCTCCGGTCACTTTTTGAAGGTCGC  900
Sbjct  1341761  GCGCACCAAAAACCGCTTGTGGACCAAAGGTGAAACCTCAGGTCACTTCCTGCAGGTGGC  1341702

Query  901      GAGCATCACGCCGGATTGTGATAACGACACCCTGCTAGTGCTGGCAAACCCGATTGGGCC  960
Sbjct  1341701  GAGCATCACACCTGATTGCGATAACGATACGCTGTTGGTGCTGGCGAATCCGATTGGGCC  1341642

Query  961      AACCTGCCACCTTGGCACCTCAAGCTGCTTCTCACCGGCTGCGCCAGATTGGGCGTTCCT  1020
Sbjct  1341641  GACCTGCCACTTGGGCACATCAAGCTGCTTCTCGCCTGCCGCACCTGATTGGGCGTTCCT  1341582

Query  1021     GTATCAGCTGGAGCAGCTGCTGGCTTCACGTAAGAGCGCCGATCCTGAGAGTTCTTACAC  1080
Sbjct  1341581  GTATCAGCTGGAGCAGCTGCTGGCTTCACGTAAGAGTGCCGATCCGGAAAGCTCTTACAC  1341522

Query  1081     CGCGAAACTCTATGCCAGCGGCACAAAGCGTATCGCGCAGAAA  1123
Sbjct  1341521  CGCAAAACTGTACGCCAGCGGCACAAAGCGCATCGCGCAGAAA  1341479
```

Range 2: 1336957 to 1338137

Score	Expect	Identities	Gaps	Strand	Frame
1098 bits(594)	0.0()	990/1186(83%)	7/1186(0%)	Plus/Minus	

Features:

```
Query  3203     TTCCGCTCATTCTCACAGCAGCACTATAATTCCAATCAATAAAATTAATCAGGCACG-AC  3261
Sbjct  1338137  TTCCGCTCATTTTCACAGCAGCACTATAATTCCAATCAATAAA-TT--TTAGGCACGAAT  1338081

Query  3262     GTTCAACGTGCCGTAGATACAGGACATCGACCTCGTCATGCAACAACAACATCATCCTCT  3321
Sbjct  1338080  CTT-TAGGTGCCGTAGAGACAGGACATCGACAACCTCATGCAACAGCAACATCATCCTCT  1338022

Query  3322     GCTAAGTGCTTCCCTCGGTACACAGCGCGAAATCGTCAGCTTCCATTTCGGTACCGATTC  3381
Sbjct  1338021  GTTGAGTGCTTCCCTTGGAACACAGCGCGAAATCGTTAGCTTCCACTTCGGTACCGATTC  1337962
```

```
Query  3382     CCAGCAGCGCGTCTACATTCAGGCGGCACTGCACGGTGATGAACTGCCGGGTATGGCGGT  3441
Sbjct  1337961  CCAGCAGCGCGTCTATATTCAGGCCGCCTTACACGGTGATGAGCTGCCCGGTATGGCGGT  1337902
Query  3442     GGCGTGGTATCTGAAACACAAGCTGCTGGCGCTGGAATCGGCCGGGCAGCTCAAATCCAA  3501
Sbjct  1337901  AGCGTGGTATTTAAAACACAAACTGCTGGCACTGGAATCCGCCGGACAGCTTAAGTCAAA  1337842
Query  3502     AATTACGCTGGTGCCGGTGGCAAATCCGCTGGCGATGGGGCAACACTGGCACGGCAGCCA  3561
Sbjct  1337841  GATCACCCTGGTGCCGGTCGCCAACCCCTTAGCGATGGGCCAGCACTGGCACGGCAGCCA  1337782
Query  3562     TTTGGGTCGATTCCATACGCTT-TCAGGTCAGGATTTCAATCGCCGTTTCCCGGCATTAG  3620
Sbjct  1337781  TCTGGGACGCTTCCATAC-CTTATCAGGTCAGGATTTCAACCGTCGCTTCCCGGCATTGG  1337723
Query  3621     GCGATACCCTGGCTGAAGAGTTGGCCGGTTCGTTGACACAGAGTGAATATGAGAACAAAC  3680
Sbjct  1337722  GTGAAACCCTGGCCGCCGAACTTGCGGATTCACTGACGCAAAGTGAGTACGAAAACAAAC  1337663
Query  3681     GGTTGATCCGTGATGCCATTGATCGCCACTATCGAGACCGCGTTGCTCGTACCGAACTGG  3740
Sbjct  1337662  GTCTGATTCGCGAAGCGATTGACCGTCATTATCGCGACCGTATTGCCAAGACTGAGCTGG  1337603
Query  3741     ATGCGCAGCGCTTCACGCTAATGCGTATGGCCAGCCAGGCAGATTTGATGATTGATCTGC  3800
Sbjct  1337602  ATTCGCAGCGCTTTACCCTGATGCGCATGGCCAGCCAGGCGGATCTCATGATCGATCTGC  1337543
Query  3801     ACTGCGACTGGGACGCATTGCCGCATCTCTACACGACCCCGCATGCGTGGCAGGATATCG  3860
Sbjct  1337542  ACTGCGACTGGGATGCACTGCCACACCTCTACACCACTCCGCATGCCTGGCAGGACATCG  1337483
Query  3861     AGCCTTTAGCGCGTTGGTTAGGTAGCGAAGTACAGCTTCTGGCCCAAATCTCTGGTGGCG  3920
Sbjct  1337482  AACCGCTGGCGCGCTGGCTAGGCAGTGAAGTGCAGCTGCTGGCGCAGATTTCAGGTGGAG  1337423
Query  3921     AGCCGTTTGATGAAGCCTGCTGCGAGCCGTGGTTAACGTTGGCCGAGCGGTTTGGTGGTG  3980
Sbjct  1337422  AGCCATTTGATGAAGCCTGCTGCGAGCCGTGGTTAACACTGGCAGAGCGCTTTGGTGATA  1337363
Query  3981     AGTATCCGATGCCACGGGGTTTGTTACCGGTGACGCTCGAATTACGTGGTGTGGCGGATG  4040
Sbjct  1337362  AGTACCCCATGCCGCGTGGATTGTTGCCAGTGACGCTGGAGTTACGCGGCGTGGCAGATG  1337303
Query  4041     TGTCGCCTGGACAAGCTGAGAAGGATGCGGATGCGATTATTAACGCGCTGATTGAAGGTG  4100
Sbjct  1337302  TTGAGCCTGAACAGGCAGAAAAAGATGCCGATGCAATTATCAATGCGCTGATTGAAGGTG  1337243
Query  4101     GCTACATTGCGGGTGACGTGGGTGAGTCCCCTGCTCTGATCAATCCTGCGACACCGCTGG  4160
Sbjct  1337242  GCTACATTGCGGGTGAGTTGGGTGAGTCCCCTGCCCTGATTAACCCAGCCACGCCGTTAG  1337183
Query  4161     CAGGTTGTGAGTATATCCATGCACCGCATTCTGGAATGCTGTTGAATCGCCGTGAGTTGG  4220
Sbjct  1337182  CAGGCTGTGAGTATATTCATGCGCCGCACTCTGGGCTGCTATTGAACCGCCGCAAGTTGG  1337123
Query  4221     GTGAGTGGATCAAGCCGGGTGAAGTGGTAGCGGAGATTGTGGACCCGATTACCGACCAGG  4280
Sbjct  1337122  GTGAGTGGATTAAGCCTGGTGAAGTGGTGGCTGAGATTGTTGACCCGATTACCGACCAGG  1337063
Query  4281     TGACGCCGCTGGTGGCAGAGTTTGGTGGGGTGCTGTATGCAAGGAACCTGATGAAGTTTG  4340
Sbjct  1337062  TGACGCCGCTGTTTGCTGAGTTTGGTGGCGTGCTGTATGCGAGGAATTTGATGAAGTTTG  1337003
Query  4341     CTACGGCTGGGATGCTGGTGGTGCGGTTGGCGGGTGAAAATGCGGG  4386
Sbjct  1337002  CGACGGCGGGGATGCTGGTGGTGAGGCTGGCTGGAGAGAATGCGGG  1336957
```

Plautia stali symbiont DNA, complete genome

Sequence ID: **AP012551.1** Length: 4035456 Number of Matches: 1

Range 1: 3902663 to 3903784

Score	Expect	Identities	Gaps	Strand	Frame
1203 bits(651)	0.0()	965/1122(86%)	0/1122(0%)	Plus/Plus	

Features:

```
Query  1        TCGTGCCACTGGCGCAGCGCTATGCGCAGGAAGGCGCTGACGAGCTGGTGTTCTATGATA  60
Sbjct  3902663  TCGTACCGCTGGCGCAGCGCTATGCCCAGGAAGGCGCTGACGAACTGGTGTTTTACGATA  3902722
Query  61       TCACCGCCTCTTCCGATGGCCGTGTGGTGGATAAAAGCTGGGTGTCACGCGTCGCAGAAG  120
Sbjct  3902723  TCACCGCCTCTTCCGACGGCCGCGTGGTGGATAAAAGCTGGGTATCGCGCGTGGCGGAAG  3902782
Query  121      TGATTGATATTCCGTTCTGCGTGGCGGGCGGTATCAAGAGTGAGGAGGATGCCGCGCGCA  180
Sbjct  3902783  TGATCGACATTCCGTTCTGCGTCGCCGGGGGCATCAAAACCCCGGAAGATGCGGCGCGCA  3902842
Query  181      TTCTGCAGTTCGGTGCGGATAAGATCTCCATCAACTCCCCGGCGCTGGCTGACCCTACGC  240
Sbjct  3902843  TTCTGCAGTTTGGCGCAGATAAGATCTCCATCAACTCACCCGCGCTGGCCGATCCGACGC  3902902
Query  241      TGATCACGCGTCTGGCAGATCGCTTTGGTGTGCAGTGTATCGTGGTGGGTATTGATACCT  300
```

```
Sbjct  3902903  TGATTACCCGGCTGGCGGATCGCTTCGGCGTGCAGTGCATCGTGGTCGGCATTGATACCT  3902962

Query  301      GGTTCGATGAAGCCACCGGCAAGTATCACGTCAATCAATATACCGGCGATGAATCACGTA  360
Sbjct  3902963  GGTTTGATGAAGCCAGCGGCAAGTATCACGTCAATCAGTACACCGGCGACGAAGCGCGCA  3903022

Query  361      CCCGTGTCACCACCTGGGAAACCCTGGATTGGGTACAGGAAGTGCAGAAGCTAGGCGCGG  420
Sbjct  3903023  CCCGCGTTACCCAGTGGGAAACACTCGACTGGGTAGAGGAAGTGCAGAAGCTGGGCGCTG  3903082

Query  421      GCGAAATCGTGCTGAACATGATGAACCAGGATGGCGTACGTAATGGCTACGATCTGGTGC  480
Sbjct  3903083  GCGAAATCGTACTGAATATGATGAACCAAGACGGCGTGCGTAACGGCTACGACCTGGTGC  3903142

Query  481      AGCTGAAGAAAGTGCGTGAGGTATGTAAAGTGCCCCTCATCGCTTCTGGCGGCGCGGGCA  540
Sbjct  3903143  AGCTGAAGAAGGTGTGTGACCTGTGTAAGGTGCCGCTGATCGCCTCCGGTGGCGCAGGGA  3903202

Query  541      CCATGCCACACTTTCTCGAAGCCTTTGAACAGGCAAACGTTGATGGCGCACTGGCCGCTT  600
Sbjct  3903203  CCATGGCGCACTTCCTCGAAGCCTTTGAGCAGGCAAACGTCGACGGCGCGCTGGCGGCTT  3903262

Query  601      CGGTGTTTCACAAACAAATTATCAATATCGGCGAGTTGAAAAACTTTCTGATCGACAACG  660
Sbjct  3903263  CGGTGTTTCACAAACAAATTATCAATATCGGCGAGTTGAAAAACTTTCTGATCGACAACG  3903322

Query  661      GTGTGGAGATTCGCGCGTGTTAACTGCAGAACAACTGGCCAAACTGGATTGGGCCAAAAC  720
Sbjct  3903323  GTGTGGAGATTCGCGCGTGTTAACTGCAGAGCAACTGTCCAGACTGGACTGGGCCAAAAC  3903382

Query  721      CGCGGGCATGATGCCCGTCATCGTCCAGCACAACGTTTCTGGCGAAGTGCTGATGCACGG  780
Sbjct  3903383  CGCGGGCATGATGCCCGTCATCGTCCAGCACAACGTCTCCGGCGAAGTGCTGATGCACGG  3903442

Query  781      TTATATGAATGAGGAAGCGCTGCAAAAGACGCTGGCGGAAGGCAATGTCACTTTCTTCTC  840
Sbjct  3903443  TTACATGAACGAGGACGCGCTGCACAAGACGCTGGCCGACGGCAATGTCACCTTCTTCTC  3903502

Query  841      TCGCACCAAAAACCGCTTGTGGACCAAGGGGGAAACCTCCGGTCACTTTTTGAAGGTCGC  900
Sbjct  3903503  CCGCACCAAAAACCGTTTATGGACCAAAGGCGAAACTTCGGACCACTTCCTCAAAGTGGT  3903562

Query  901      GAGCATCACGCCGGATTGTGATAACGACACCCTGCTAGTGCTGGCAAACCCGATTGGGCC  960
Sbjct  3903563  TAGCATTACGCCAGATTGCGATAACGACACGCTGCTGGTGCTGGCCAATCCGATTGGCCC  3903622

Query  961      AACCTGCCACCTTGGCACCTCAAGCTGCTTCTCACCGGCTGCGCCAGATTGGGCGTTCCT  1020
Sbjct  3903623  CACCTGCCATCTCGGTACCTCGAGCTGCTTTTCGCCTGCCGCACCCGACTGGACGTTCCT  3903682

Query  1021     GTATCAGCTGGAGCAGCTGCTGGCTTCACGTAAGAGCGCCGATCCTGAGAGTTCTTACAC  1080
Sbjct  3903683  GTATCAGCTGGAGCAGTTGCTGGCCTCGCGTAAAAGTGCCGATCCCGCCAGCTCCTATAC  3903742

Query  1081     CGCGAAACTCTATGCCAGCGGCACAAAGCGTATCGCGCAGAA  1122
Sbjct  3903743  GGCGAAGCTGTACGCCAGCGGCACCAAGCGCATCGCACAGAA  3903784
```

Pantoea sp. At-9b, complete genome

Sequence ID: **CP002433.1** Length: 4368708 Number of Matches: 1

Range 1: 2804260 to 2805382

Score	Expect	Identities	Gaps	Strand	Frame
1149 bits(622)	0.0()	958/1125(85%)	4/1125(0%)	Plus/Plus	

Features:

```
Query  1        TCGTGCCACTGGCGCAGCGCTATGCGCAGGAAGGCGCTGACGAGCTGGTGTTCTATGATA  60
Sbjct  2804260  TCGTGCCACTGGCGCAACGTTATGCGCAGGAAGGGGCGGATGAACTGGTGTTTTATGATA  2804319

Query  61       TCACCGCCTCTTCCGATGGCCGTGTGGTGGATAAAAGCTGGGTGTCACGCGTCGCAGAAG  120
Sbjct  2804320  TCACCGCCTCGTCTGATGGCCGTGTCGTGGATAAAAGCTGGGTCTCGCGTGTGGCGGAAG  2804379

Query  121      TGATTGATATTCCGTTCTGCGTGGCGGGCGGTATCAAGAGTGAGGAGGATGCCGCGCGCA  180
Sbjct  2804380  TGATTGATATTCCTTTCTGTGTAGCAGGGGGAATCAAGAGCGAAGCGGATGCCGCGCGCA  2804439

Query  181      TTCTGCAGTTCGGTGCGGATAAGATCTCCATCAACTCCCCGGCGCTGGCTGA-CCCTACG  239
Sbjct  2804440  TTCTGGAATTTGGTGCGGATAAGATCTCCATCAACTCACCCGCGCTGGCTGATCCCT-CG  2804498

Query  240      CTGATCACGCGTCTGGCAGATCGCTTTGGTGTGCAGTGTATCGTGGTGGGTATTGATACC  299
Sbjct  2804499  CTGATTACCCGTCTGGCCGATCGCTTTGGCGTGCAATGTATTGTGGTGGGGATCGATACC  2804558

Query  300      TGGTTCGATGAAGCCACCGGCAAGTATCACGTCAATCAATATACCGGCGATGAATCACGT  359
Sbjct  2804559  TGGTTTGACGACGCGACCGGCAAATATCACGTTAATCAGTATACCGGTGATGAATCCCGC  2804618

Query  360      ACCCGTGTCAC-CACCTGGGAAACCCTGGATTGGGTACAGGAAGTGCAGAAGCTAGGCGC  418
```

```
Sbjct  2804619   ACCCGTGTGACGCA-ATGGGAAACCCTGGATTGGGTGCAGGAAGTGCAGAAGCTGGGCGC  2804677

Query  419       GGGCGAAATCGTGCTGAACATGATGAACCAGGATGGCGTACGTAATGGCTACGATCTGGT  478
Sbjct  2804678   GGGCGAAATCGTTCTCAATATGATGAACCAGGACGGCGTGCGCAACGGTTATGACCTGGT  2804737

Query  479       GCAGCTGAAGAAAGTGCGTGAGGTATGTAAAGTGCCCCTCATCGCTTCTGGCGGCGCGGG  538
Sbjct  2804738   ACAGCTGAAGAAAGTCCGCGATGTCTGTAAGGTACCGTTGATTGCTTCCGGTGGCGCTGG  2804797

Query  539       CACCATGCCACACTTTCTCGAAGCCTTTGAACAGGCAAACGTTGATGGCGCACTGGCCGC  598
Sbjct  2804798   CACCATGGCACACTTCCTCGAAGCGTTTGAACAAGCTAACGTCGATGGCGCGCTGGCGGC  2804857

Query  599       TTCGGTGTTTCACAAACAAATTATCAATATCGGCGAGTTGAAAAACTTTCTGATCGACAA  658
Sbjct  2804858   ATCGGTGTTTCACAAACAGATTATTAATATCGGCGAGCTGAAAAGCTTCCTGATTGACAA  2804917

Query  659       CGGTGTGGAGATTCGCGCGTGTTAACTGCAGAACAACTGGCCAAACTGGATTGGGCCAAA  718
Sbjct  2804918   CGGTGTGGAGATTCGCGCGTGTTAACTGCAGAACAACTGACCCGTCTGGATTGGGTCAAA  2804977

Query  719       ACCGCGGGCATGATGCCCGTCATCGTCCAGCACAACGTTTCTGGCGAAGTGCTGATGCAC  778
Sbjct  2804978   ACCGCGGGCATGATGCCCGTTATCGTCCAGCACAACGTCTCTGGCGAAGTGCTGATGCAC  2805037

Query  779       GGTTATATGAATGAGGAAGCGCTGCAAAAGACGCTGGCGGAAGGCAATGTCACTTTCTTC  838
Sbjct  2805038   GGTTATATGAACGAGGAAGCGTTGCAGAAAACCCTCGCGGAGGGCAATGTCACCTTCTTC  2805097

Query  839       TCTCGCACCAAAAACCGCTTGTGGACCAAGGGGGAAACCTCCGGTCACTTTTTGAAGGTC  898
Sbjct  2805098   TCGCGCACCAAAAATCGCCTGTGGACCAAAGGTGAAACCTCAGGCCACTTCCTGAAAGTG  2805157

Query  899       GCGAGCATCACGCCGGATTGTGATAACGACACCCTGCTAGTGCTGGCAAACCCGATTGGG  958
Sbjct  2805158   GTGAGCATTACTCCCGATTGTGACAACGATACGCTGCTGGTGCTGGCGAACCCGATTGGC  2805217

Query  959       CCAACCTGCCACCTTGGCACCTCAAGCTGCTTCTCACCGGCTGCGCCAGATTGGGCGTTC  1018
Sbjct  2805218   CCCACCTGCCATCTCGGTACTTCCAGTTGTTTCTCTCCGGCAGCACCGGACTGGACTTTC  2805277

Query  1019      CTGTATCAGCTGGAGCAGCTGCTGGCTTCACGTAAGAGCGCCGATCCTGAGAGTTCTTAC  1078
Sbjct  2805278   CTCTATCAGTTGGAACAACTGCTGGCGGAGCGTAAAAGTGCTGACCCCGCCAGCTCCTAC  2805337

Query  1079      ACCGCGAAACTCTATGCCAGCGGCACAAAGCGTATCGCGCAGAAA  1123
Sbjct  2805338   ACCGCGAAGTTGTATGCCAGCGGCACCAAGCGTATCGCACAGAAA  2805382
```

Pantoea sp. PSNIH1, complete genome

Sequence ID: **CP009880.2** Length: 3488376 Number of Matches: 1

Range 1: 3324633 to 3325752

Score	Expect	Identities	Gaps	Strand	Frame
1077 bits(583)	0.0()	943/1122(84%)	4/1122(0%)	Plus/Plus	

Features:

```
Query  3         GTGCCACTGGCGCAGCGCTATGCGCAGGAAGGCGCTGACGAGCTGGTGTTCTATGATATC  62
Sbjct  3324633   GTGCCGCTGGCGCAGCGCTACGCGCAGGAAGGGGCCGACGAGCTGGTGTTTTATGACATC  3324692

Query  63        ACCGCCTCTTCCGATGGCCGTGTGGTGGATAAAAGCTGGGTGTCACGCGTCGCAGAAGTG  122
Sbjct  3324693   ACCGCCTCATCGGATGGCCGCGTGGTCGACAAAAGCTGGGTCTCCCGCGTAGCGGAAGTG  3324752

Query  123       ATTGATATTCCGTTCTGCGTGGCGGGCGGTATCAAGAGTGAGGAGGATGCCGCGCGCATT  182
Sbjct  3324753   ATCGACATTCCGTTCTGCGTCGCGGGCGGCATCAGAAGCGAGGAGGATGCCGCGCGCATT  3324812

Query  183       CTGCAGTTCGGTGCGGATAAGATCTCCATCAACTCCCCGGCGCTGGCTGACCCTACGCTG  242
Sbjct  3324813   CTGCAGTTTGGCGCTGACAAAATATCTGTCAACTCCCCGGCGCTGGCCGATCCGACGCTG  3324872

Query  243       ATCACGCGTCTGGCAGATCGCTTTGGTGTGCAGTGTATCGTGGTGGGTATTGATACCTGG  302
Sbjct  3324873   ATCACCCGTCTCGCCGACCGTTTTGGCGTGCAGTGCATAGTAGTGGGTATCGATACTTGG  3324932

Query  303       TTCGATGAAGCCACCGGCAAGTATCACGTCAATCAATATACCGGCGATGAATCACGTACC  362
Sbjct  3324933   TTTGATGAGGCGAGCGGTAAATATCACGTTAACCAGTATACCGGTGATGAAGCGCGTACC  3324992

Query  363       CGTGTCAC-CACCTGGGAAACCCTGGATTGGGTACAGGAAGTGCAGAAGCTAGGCGCGGG  421
Sbjct  3324993   CGGGTAACGCA-GTGGGAAACCCTTGACTGGGTGCGGGAAGTGCAGAAGCTCGGCGCGGG  3325051

Query  422       CGAAATCGTGCTGAACATGATGAACCAGGATGGCGTACGTAATGGCTACGATCTGGTGCA  481
Sbjct  3325052   CGAGATCGTGCTGAATATGATGAATCAGGATGGCGTACGTAACGGCTACGATCTGGTTCA  3325111

Query  482       GCTGAAGAAAGTGCGTGAGGTATGTAAAGTGCCCCTCATCGCTTCTGGCGGCGCGGGCAC  541
Sbjct  3325112   GCTGCAGAAGGTCCGCGAAGTGTGCAGCGTGCCGCTAATCGCGTCCGGCGGTGCCGGCAC  3325171
```

```
Query  542      CATGCCACACTTTCTCGAAGCCTTTGAACAGGCAAACGTTGATGGCGCACTGGCCGCTTC  601
                |||||| |||||| || || ||||||   ||||||||||||||||| || ||||| || ||
Sbjct  3325172  CATGCCGCACTTCCTTGACGCCTTTACGCAGGCAAACGTTGATGGTGCCCTGGCGGCGTC  3325231

Query  602      GGTGTTTCACAAACAAATTATCAATATCGGCGAGTTGAAAAACTTTCTGATCGACAACGG  661
                |||||| || || || |||| |||||| | ||| ||||||||||||||||||||||||
Sbjct  3325232  GGTGTTCCATAAGCAGATTATTAATATCAGTGAGCTGAAAAACTTTCTGATCGACAACGG  3325291

Query  662      TGTGGAGATTCGCGCGTGTTAACTGCAGAACAACTGGCCAAACTGGATTGGGCCAAAACC  721
                ||||||||||||||||||||||| ||||| |||||||||   ||||| |||| |||||||
Sbjct  3325292  TGTGGAGATTCGCGCGTGTTAACAGCAGACCAACTGGCCCGCCTGGACTGGGTCAAAACC  3325351

Query  722      GCGGGCATGATGCCCGTCATCGTCCAGCACAACGTTTCTGGCGAAGTGCTGATGCACGGT  781
                ||||||||||||||||| ||||| |||||||||||| || ||||||||||||||||||||
Sbjct  3325352  GCGGGCATGATGCCCGTTATCGTGCAGCACAACGTCTCCGGCGAAGTGCTGATGCACGGT  3325411

Query  782      TATATGAATGAGGAAGCGCTGCAAAAGACGCTGGCGGAAGGCAATGTCACTTTCTTCTCT  841
                |||||||||||||  |||||| |||| |||||||||||||||  |||| || ||||| 
Sbjct  3325412  TATATGAATGAGGCGGCGCTGGAAAAAACGCTGGCGGAAGGCAACGTCACCTTTTTCTCG  3325471

Query  842      CGCACCAAAAACCGCTTGTGGACCAAGGGGGAAACCTCCGGTCACTTTTTGAAGGTCGCG  901
                ||||||||| | ||  | |||||||| || ||| |||||||| |||||| || | | |
Sbjct  3325472  CGCACCAAAGAGCGGCTCTGGACCAAAGGCGAATCCTCCGGCCACTTTCTGCAAGTGGTG  3325531

Query  902      AGCATCACGCCGGATTGTGATAACGACACCCTGCTAGTGCTGGCAAACCCGATTGGGCCA  961
                ||||| ||||| || |||||||||| || ||||| |||||||| ||||| || || || 
Sbjct  3325532  AGCATTACGCCAGACTGTGATAACGATACGCTGCTGGTGCTGGCGAACCCCATCGGACCG  3325591

Query  962      ACCTGCCACCTTGGCACCTCAAGCTGCTTCTCACCGGCTGCGCCAGATTGGGCGTTCCTG  1021
                || ||||| ||||||||||| ||||||||||| || || || || || ||| ||||||| 
Sbjct  3325592  ACGTGCCATCTTGGCACCTCCAGCTGCTTCTCGCCTGCCGCCCCGGAGTGGACGTTCCTC  3325651

Query  1022     TATCAGCTGGAGCAGCTGCTGGCTTCACGTAA-GAGCGCCGATCCTGAGAGTTCTTACAC  1080
                ||||| ||||||||||||||||| || || || ||  |||||||| |  || || || ||
Sbjct  3325652  TATCAACTGGAGCAGCTGCTGGCGTCGCGGAAAGAT-GCCGATCCCGCCAGCTCCTATAC  3325710

Query  1081     CGCGAAACTCTATGCCAGCGGCACAAAGCGTATCGCGCAGAA  1122
                ||| |  ||||| ||||||||||| ||||||||| ||||||||
Sbjct  3325711  CGCCAGCCTCTACGCCAGCGGCACCAAGCGTATTGCGCAGAA  3325752
```

BLAST is a registered trademark of the National Library of Medicine

Support center Mailing list YouTube

- National Library Of Medicine
- National Institutes Of Health
- U.S. Department of Health & Human Services
- USA.gov

NCBI

National Center for Biotechnology Information, U.S. National Library of Medicine 8600 Rockville Pike, Bethesda MD, 20894 USA

Policies and Guidelines | Contact
