# Supplementary material for: Grafting or pruning in the animal tree: lateral gene transfer and gene loss?
Source: BMC Genomics. 2018 Jun 18;19:470. doi: 10.1186/s12864-018-4832-5 (PMC6006793; doi:10.1186/s12864-018-4832-5)
Supplement: Supplementary file 4 — BLASTX search of A. pisum strain LSR1 unplaced genomic scaffold, Acyr_2.0 Scaffold2139 (NW_003385628.1) against NR allowing for 20,000 matches with an e-value below 0.00001. (PDF 269 kb) [file 12864_2018_4832_MOESM4_ESM.pdf]

BLAST ® » blastx » RID-9JRXS99V015

BLAST Results

Questions/comments

Job title: ref|NW_003385628.1| (7613 letters)

RID 9JRXS99V015 (Expires on 03-03 21:29 pm)

**Query ID**	NW_003385628.1	**Database Name**	nr
**Description**	Acyrthosiphon pisum strain LSR1 unplaced genomic scaffold, Acyr_2.0 Scaffold2139	**Description**	All non-redundant GenBank CDS translations+PDB+SwissProt+PIR+PRF excluding environmental samples from WGS projects
**Molecule type**	dna	**Program**	BLASTX 2.8.0+
**Query Length**	7613		

Graphic Summary

Putative conserved domains have been detected, click on the image below for detailed results.

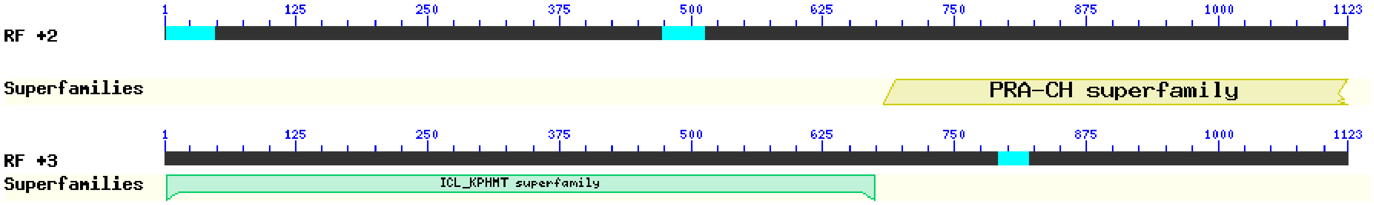

Distribution of the top 103 Blast Hits on 100 subject sequences

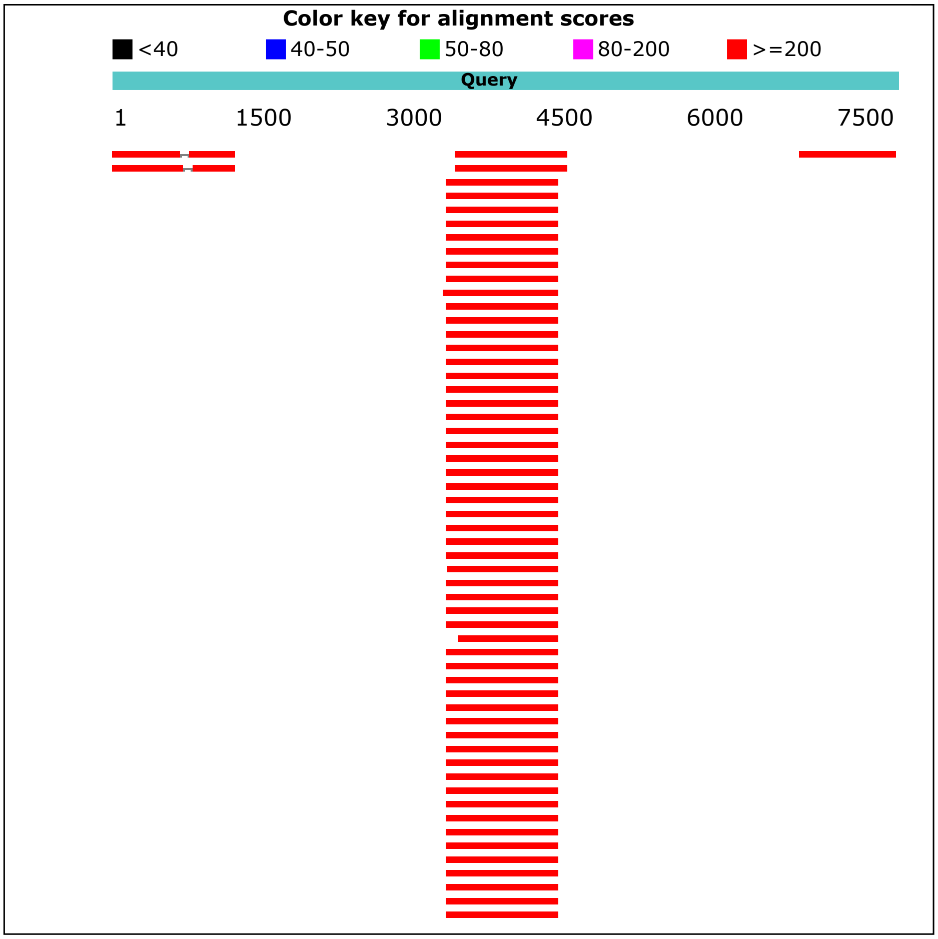

Descriptions

Sequences producing significant alignments:

Description	Max score	Total score	Query cover	E value	Ident	Accession
succinylglutamate desuccinylase [Type-E symbiont of Plautia stali]	746	746	14%	0.0	100%	WP_058962033.1
succinylglutamate desuccinylase [Enterobacter cancerogenus]	742	742	14%	0.0	99%	WP_034824751.1
succinylglutamate desuccinylase [Pantoea sp. GL120224-02]	739	739	14%	0.0	99%	WP_097116667.1
succinylglutamate desuccinylase [Pantoea sp. BL1]	736	736	14%	0.0	99%	WP_045832306.1
succinylglutamate desuccinylase [Pantoea sp. SM3]	736	736	14%	0.0	98%	WP_045815228.1
succinylglutamate desuccinylase [Pantoea rwandensis]	733	733	14%	0.0	98%	WP_038646005.1
succinylglutamate desuccinylase [Pantoea rodasii]	732	732	14%	0.0	98%	WP_100700970.1
succinylglutamate desuccinylase [Pantoea rwandensis]	724	724	14%	0.0	96%	WP_084935080.1
MULTISPECIES: succinylglutamate desuccinylase [Pantoea]	719	719	14%	0.0	96%	WP_039327834.1
succinylglutamate desuccinylase [Type-D symbiont of Plautia stali]	717	717	14%	0.0	95%	WP_058973522.1
putative deacylase [Pantoea sp. GM01]	697	697	14%	0.0	91%	EJL89595.1
succinylglutamate desuccinylase [Pantoea dispersa]	694	694	14%	0.0	93%	WP_058771659.1
succinylglutamate desuccinylase [Type-C symbiont of Plautia stali]	694	694	14%	0.0	92%	WP_059011591.1
MULTISPECIES: hypothetical protein [Pantoea]	693	693	14%	0.0	92%	WP_008104837.1
succinylglutamate desuccinylase [Pantoea sp. 596]	692	692	14%	0.0	93%	WP_101763384.1
succinylglutamate desuccinylase [Enterobacter ludwigii]	691	691	14%	0.0	92%	WP_061717715.1
succinylglutamate desuccinylase [Pantoea dispersa]	690	690	14%	0.0	91%	WP_058757309.1
succinylglutamate desuccinylase [Pantoea sp. RIT-PI-b]	690	690	14%	0.0	92%	WP_049852474.1
succinylglutamate desuccinylase [Pantoea dispersa]	689	689	14%	0.0	91%	WP_058777966.1
succinylglutamate desuccinylase [Pantoea sp. GM01]	689	689	14%	0.0	92%	WP_036620808.1
succinylglutamate desuccinylase [Pantoea dispersa]	689	689	14%	0.0	91%	WP_031280067.1
succinylglutamate desuccinylase [Pantoea sp. VS1]	688	688	14%	0.0	91%	WP_088518039.1
succinylglutamate desuccinylase [Enterobacter ludwigii]	688	688	14%	0.0	92%	WP_064739211.1
succinylglutamate desuccinylase [Pantoea sp. ICBG 828]	687	687	14%	0.0	91%	WP_104189698.1
succinylglutamate desuccinylase [Pantoea dispersa]	686	686	14%	0.0	90%	WP_058782008.1
succinylglutamate desuccinylase [Pantoea wallisii]	684	684	14%	0.0	91%	ORM72928.1
MULTISPECIES: succinylglutamate desuccinylase [unclassified Enterobacteriaceae (miscellaneous)]	684	684	14%	0.0	91%	WP_097096734.1
hypothetical protein [Pantoea sp. A4]	678	678	14%	0.0	88%	WP_017346595.1
succinylglutamate desuccinylase [Pantoea cypripedii]	677	677	14%	0.0	90%	WP_084875982.1
hypothetical protein [Pantoea sp. At-9b]	676	676	14%	0.0	89%	WP_013509729.1
hypothetical protein N172_10500 [Pantoea dispersa EGD-AAK13]	672	672	14%	0.0	91%	ERH62572.1
succinylglutamate desuccinylase/aspartoacylase [Pantoea sp. AS-PWVM4]	670	670	14%	0.0	88%	WP_021185958.1
succinylglutamate desuccinylase [Pantoea eucrina]	669	669	14%	0.0	87%	WP_065648935.1
MULTISPECIES: succinylglutamate desuccinylase [Pantoea]	666	666	14%	0.0	87%	WP_039385336.1
succinylglutamate desuccinylase [Pantoea sp. BRM17]	662	662	14%	0.0	86%	PPS58720.1
succinylglutamate desuccinylase [Pantoea dispersa]	602	602	12%	0.0	91%	WP_058776718.1
hypothetical protein [Type-E symbiont of Plautia stali]	594	594	12%	0.0	99%	WP_058962031.1
hypothetical protein [Pantoea vagans]	592	592	14%	0.0	71%	WP_033733219.1
hypothetical protein [Pantoea agglomerans]	587	587	14%	0.0	71%	WP_069025174.1
hypothetical protein [Pantoea vagans]	587	587	14%	0.0	71%	WP_083069020.1
hypothetical protein [Pantoea agglomerans]	587	587	14%	0.0	70%	WP_039391543.1

Description	Max score	Total score	Query cover	E value	Ident	Accession
hypothetical protein [Pantoea sp. MBLJ3]	586	586	14%	0.0	71%	WP_039659817.1
hypothetical protein AL522_16695 [Pantoea vagans]	585	585	14%	0.0	70%	AMG59131.1
hypothetical protein [Pantoea agglomerans]	584	584	14%	0.0	70%	WP_004571306.1
MULTISPECIES: hypothetical protein [Pantoea]	583	583	14%	0.0	71%	WP_033758378.1
hypothetical protein [Pantoea agglomerans]	583	583	14%	0.0	70%	WP_089414012.1
MULTISPECIES: hypothetical protein [Pantoea]	583	583	14%	0.0	70%	WP_033788292.1
hypothetical protein [Pantoea septica]	583	583	14%	0.0	69%	WP_033791523.1
hypothetical protein [Pantoea agglomerans]	583	583	14%	0.0	70%	WP_031591336.1
hypothetical protein [Pantoea sp. SL1_M5]	583	583	14%	0.0	71%	WP_010248080.1
hypothetical protein [Pantoea agglomerans]	582	582	14%	0.0	70%	WP_033767791.1
hypothetical protein [Pantoea agglomerans]	582	582	14%	0.0	70%	WP_086906082.1
hypothetical protein [Pantoea agglomerans]	581	581	14%	0.0	70%	WP_064703707.1
hypothetical protein [Pantoea agglomerans]	580	580	14%	0.0	70%	WP_098052867.1
hypothetical protein [Pantoea ananatis]	580	580	14%	0.0	70%	WP_045140284.1
hypothetical protein [Pantoea sp. aB]	580	580	14%	0.0	70%	WP_008926065.1
hypothetical protein [Pantoea agglomerans]	579	579	14%	0.0	70%	WP_010670966.1
hypothetical protein [Pantoea sp. OV426]	579	579	14%	0.0	70%	WP_090959634.1
hypothetical protein [Pantoea sp. 9140]	579	579	14%	0.0	70%	WP_033783636.1
hypothetical protein [Pantoea vagans]	578	578	14%	0.0	70%	WP_095706914.1
hypothetical protein [Pantoea vagans]	577	577	14%	0.0	70%	WP_013358444.1
hypothetical protein HA42_02255 [Pantoea deleyi]	555	555	14%	3e-175	66%	ORM85645.1
hypothetical protein [Pantoea agglomerans]	553	553	14%	8e-175	67%	WP_064690207.1
Histidine biosynthesis bifunctional protein hisB [Beauveria bassiana D1-5]	397	644	13%	9e-174	91%	KGQ14013.1
hypothetical protein BXA16_20965 [Salmonella enterica subsp. enterica serovar Typhimurium]	409	637	13%	1e-171	89%	ONF75263.1
hypothetical protein [Pantoea sp. FDAARGOS_194]	543	543	14%	4e-171	66%	WP_096073456.1
hypothetical protein [Pantoea septica]	540	540	14%	7e-170	67%	WP_084883088.1
hypothetical protein [Pantoea vagans]	536	536	14%	1e-168	66%	WP_048784169.1
succinylglutamate desuccinylase [Izhakiella australiensis]	533	533	14%	2e-168	71%	WP_078002897.1
hypothetical protein [Pantoea sp. ICBG 985]	535	672	14%	3e-168	93%	WP_104093136.1
hypothetical protein [Pantoea sp. NGS-ED-1003]	535	535	14%	3e-168	66%	WP_033750138.1
succinylglutamate desuccinylase [Erwinia billingiae]	530	530	14%	2e-167	71%	WP_053142109.1
hypothetical protein [Pantoea anthophila]	530	530	14%	3e-166	64%	WP_046102184.1
succinylglutamate desuccinylase/aspartoacylase [Erwinia billingiae]	526	526	14%	5e-166	70%	WP_013202934.1
succinylglutamate desuccinylase [Izhakiella capsodis]	524	524	14%	4e-165	67%	WP_092877593.1
hypothetical protein [Pantoea sp. Sc1]	526	526	14%	7e-165	64%	WP_009089300.1
hypothetical protein [Type-F symbiont of Plautia stali]	525	525	14%	7e-165	64%	WP_058956677.1
succinylglutamate desuccinylase [Pluralibacter gergoviae]	521	521	14%	2e-164	69%	WP_043084670.1
succinylglutamate desuccinylase [Pluralibacter gergoviae]	521	521	14%	4e-164	69%	WP_045287969.1
succinylglutamate desuccinylase [Pluralibacter gergoviae]	520	520	14%	6e-164	69%	WP_053516180.1
succinylglutamate desuccinylase [Pluralibacter gergoviae]	520	520	14%	1e-163	69%	WP_048286765.1
succinylglutamate desuccinylase [Pluralibacter gergoviae]	520	520	14%	1e-163	69%	WP_086497882.1
succinylglutamate desuccinylase [Pluralibacter gergoviae]	519	519	14%	2e-163	69%	WP_098940203.1
succinylglutamate desuccinylase [Pluralibacter gergoviae]	518	518	14%	5e-163	69%	WP_048280343.1
succinylglutamate desuccinylase [Pluralibacter gergoviae]	516	516	14%	1e-162	69%	WP_071198849.1
succinylglutamate desuccinylase [Pluralibacter gergoviae]	516	516	14%	2e-162	69%	WP_048285970.1

Description	Max score	Total score	Query cover	E value	Ident	Accession
succinylglutamate desuccinylase [Pluralibacter gergoviae]	516	516	14%	2e-162	69%	WP_048273800.1
succinylglutamate desuccinylase [Pluralibacter gergoviae]	516	516	14%	3e-162	69%	WP_048282576.1
succinylglutamate desuccinylase [Pluralibacter gergoviae]	515	515	14%	4e-162	69%	WP_048252418.1
succinylglutamate desuccinylase [Erwinia typographi]	507	507	14%	2e-159	67%	WP_034887669.1
succinylglutamate desuccinylase [Erwinia mallotivora]	505	505	14%	2e-158	69%	WP_034937737.1
succinylglutamate desuccinylase [Erwinia tasmaniensis]	503	503	14%	7e-158	67%	WP_012441086.1
succinylglutamate desuccinylase/aspartoacylase [Erwinia pyrifoliae]	499	499	14%	3e-156	67%	WP_012667731.1
succinylglutamate desuccinylase [Erwinia sp. ErVv1]	498	498	14%	5e-156	70%	WP_067708392.1
succinylglutamate desuccinylase [Erwinia sp. Ejp617]	497	497	14%	1e-155	66%	WP_014544593.1
succinylglutamate desuccinylase [Gibbsiella quercinecans]	490	490	14%	3e-153	66%	WP_095845068.1
hypothetical protein [Erwinia piriflorinigrans]	485	485	14%	1e-151	67%	WP_023655470.1
hypothetical protein [Pantoea stewartii]	487	487	14%	2e-151	60%	WP_033740451.1
hypothetical protein [Pantoea stewartii]	487	487	14%	2e-151	60%	WP_039338115.1
hypothetical protein [Pantoea stewartii]	487	487	14%	3e-151	60%	WP_058708397.1

Alignments

succinylglutamate desuccinylase [Type-E symbiont of Plautia stali]
Sequence ID: **WP_058962033.1** Length: 371 Number of Matches: 1
Range 1: 1 to 363

Score	Expect	Method	Identities	Positives	Gaps	Frame
746 bits(1926)	0.0()	Compositional matrix adjust.	363/363(100%)	363/363(100%)	0/363(0%)	+2

Features:

```
Query  3299   MQQQHHPLLSASLGTQREIVSFHFGTDSQQRVYIQAALHGDELPGMAVAWYLKHKLLALE  3478
              MQQQHHPLLSASLGTQREIVSFHFGTDSQQRVYIQAALHGDELPGMAVAWYLKHKLLALE
Sbjct  1      MQQQHHPLLSASLGTQREIVSFHFGTDSQQRVYIQAALHGDELPGMAVAWYLKHKLLALE  60

Query  3479   SAGQLKSKITLVPVANPLAMGQHWHGSHLGRFHTLSGQDFNRRFPALGDTLAEELAGSLT  3658
              SAGQLKSKITLVPVANPLAMGQHWHGSHLGRFHTLSGQDFNRRFPALGDTLAEELAGSLT
Sbjct  61     SAGQLKSKITLVPVANPLAMGQHWHGSHLGRFHTLSGQDFNRRFPALGDTLAEELAGSLT  120

Query  3659   QSEYENKRLIRDAIDRHYRDRVARTELDAQRFTLMRMASQADLMIDLHCDWDALPHLYTT  3838
              QSEYENKRLIRDAIDRHYRDRVARTELDAQRFTLMRMASQADLMIDLHCDWDALPHLYTT
Sbjct  121    QSEYENKRLIRDAIDRHYRDRVARTELDAQRFTLMRMASQADLMIDLHCDWDALPHLYTT  180

Query  3839   PHAWQDIEPLARWLGSEVQLLAQISGGEPFDEACCEPWLTLAERFGGEYPMPRGLLPVTL  4018
              PHAWQDIEPLARWLGSEVQLLAQISGGEPFDEACCEPWLTLAERFGGEYPMPRGLLPVTL
Sbjct  181    PHAWQDIEPLARWLGSEVQLLAQISGGEPFDEACCEPWLTLAERFGGEYPMPRGLLPVTL  240

Query  4019   ELRGVADVSPGQAEKDADAIINALIEGGYIAGDVGESPALINPATPLAGCEYIHAPHSGM  4198
              ELRGVADVSPGQAEKDADAIINALIEGGYIAGDVGESPALINPATPLAGCEYIHAPHSGM
Sbjct  241    ELRGVADVSPGQAEKDADAIINALIEGGYIAGDVGESPALINPATPLAGCEYIHAPHSGM  300

Query  4199   LLNRRELGEWIKPGEVVAEIVDPITDQVTPLVAEFGGVLYARNLMKFATAGMLVVRLAGE  4378
              LLNRRELGEWIKPGEVVAEIVDPITDQVTPLVAEFGGVLYARNLMKFATAGMLVVRLAGE
Sbjct  301    LLNRRELGEWIKPGEVVAEIVDPITDQVTPLVAEFGGVLYARNLMKFATAGMLVVRLAGE  360

Query  4379   NAG  4387
              NAG
Sbjct  361    NAG  363
```

succinylglutamate desuccinylase [Enterobacter cancerogenus]
Sequence ID: **WP_034824751.1** Length: 371 Number of Matches: 1

See 1 more title(s)
Range 1: 1 to 363

Score	Expect	Method	Identities	Positives	Gaps	Frame
742 bits(1916)	0.0()	Compositional matrix adjust.	361/363(99%)	362/363(99%)	0/363(0%)	+2

Features:

```
Query  3299   MQQQHHPLLSASLGTQREIVSFHFGTDSQQRVYIQAALHGDELPGMAVAWYLKHKLLALE  3478
              MQQQHHPLLSASLGTQREIVSFHFGTDSQQRVYIQAALHGDELPGMAVAWYLKHKLLALE
Sbjct  1      MQQQHHPLLSASLGTQREIVSFHFGTDSQQRVYIQAALHGDELPGMAVAWYLKHKLLALE  60

Query  3479   SAGQLKSKITLVPVANPLAMGQHWHGSHLGRFHTLSGQDFNRRFPALGDTLAEELAGSLT  3658
              SAGQLKSKITLVPVANPLAMGQHWHGSHLGRFHTLSGQDFNRRFPALGDTLAEELAGSLT
Sbjct  61     SAGQLKSKITLVPVANPLAMGQHWHGSHLGRFHTLSGQDFNRRFPALGDTLAEELAGSLT  120
```

```
Query  3659  QSEYENKRLIRDAIDRHYRDRVARTELDAQRFTLMRMASQADLMIDLHCDWDALPHLYTT  3838
             QSEYENKRLIRDAIDRHYRDRVARTELDAQRFTLMRMASQADLMIDLHCDWDALPHLYTT
Sbjct  121   QSEYENKRLIRDAIDRHYRDRVARTELDAQRFTLMRMASQADLMIDLHCDWDALPHLYTT  180

Query  3839  PHAWQDIEPLARWLGSEVQLLAQISGGEPFDEACCEPWLTLAERFGGEYPMPRGLLPVTL  4018
             PHAWQDIEPLARWLGSEVQLLAQISGGEPFDEACCEPWLTLAERFG EYPMPRGLLPVTL
Sbjct  181   PHAWQDIEPLARWLGSEVQLLAQISGGEPFDEACCEPWLTLAERFGSEYPMPRGLLPVTL  240

Query  4019  ELRGVADVSPGQAEKDADAIINALIEGGYIAGDVGESPALINPATPLAGCEYIHAPHSGM  4198
             ELRGVADVSPGQAEKDADAIINALIEGGYIAGDVGESPALINPATPLAGCEYIHAPHSG+
Sbjct  241   ELRGVADVSPGQAEKDADAIINALIEGGYIAGDVGESPALINPATPLAGCEYIHAPHSGL  300

Query  4199  LLNRRELGEWIKPGEVVAEIVDPITDQVTPLVAEFGGVLYARNLMKFATAGMLVVRLAGE  4378
             LLNRRELGEWIKPGEVVAEIVDPITDQVTPLVAEFGGVLYARNLMKFATAGMLVVRLAGE
Sbjct  301   LLNRRELGEWIKPGEVVAEIVDPITDQVTPLVAEFGGVLYARNLMKFATAGMLVVRLAGE  360

Query  4379  NAG  4387
             NAG
Sbjct  361   NAG  363
```

succinylglutamate desuccinylase [Pantoea sp. GL120224-02]

Sequence ID: **WP_097116667.1** Length: 371 Number of Matches: 1

See 1 more title(s)

Range 1: 1 to 363

Score	Expect	Method	Identities	Positives	Gaps	Frame
739 bits(1908)	0.0()	Compositional matrix adjust.	359/363(99%)	362/363(99%)	0/363(0%)	+2

Features:

```
Query  3299  MQQQHHPLLSASLGTQREIVSFHFGTDSQQRVYIQAALHGDELPGMAVAWYLKHKLLALE  3478
             MQQQHHPLLSASLGTQREIVSFHFGTDSQQRVYIQAALHGDELPGMAVAWYLKH+LLALE
Sbjct  1     MQQQHHPLLSASLGTQREIVSFHFGTDSQQRVYIQAALHGDELPGMAVAWYLKHRLLALE  60

Query  3479  SAGQLKSKITLVPVANPLAMGQHWHGSHLGRFHTLSGQDFNRRFPALGDTLAEELAGSLT  3658
             SAGQLKSKITLVPVANPLAMGQHWHGSHLGRFHTLSGQDFNRRFPALGDTLAEELAGSLT
Sbjct  61    SAGQLKSKITLVPVANPLAMGQHWHGSHLGRFHTLSGQDFNRRFPALGDTLAEELAGSLT  120

Query  3659  QSEYENKRLIRDAIDRHYRDRVARTELDAQRFTLMRMASQADLMIDLHCDWDALPHLYTT  3838
             QSEYENKRLIRDAIDRHYRDRVARTELDAQRFTLMRMASQADLMIDLHCDWDALPH+YTT
Sbjct  121   QSEYENKRLIRDAIDRHYRDRVARTELDAQRFTLMRMASQADLMIDLHCDWDALPHIYTT  180

Query  3839  PHAWQDIEPLARWLGSEVQLLAQISGGEPFDEACCEPWLTLAERFGGEYPMPRGLLPVTL  4018
             PHAWQDIEPLARWLGSEVQLLAQISGGEPFDEACCEPWLTLAERFGGEYPMPRGLLPVTL
Sbjct  181   PHAWQDIEPLARWLGSEVQLLAQISGGEPFDEACCEPWLTLAERFGGEYPMPRGLLPVTL  240

Query  4019  ELRGVADVSPGQAEKDADAIINALIEGGYIAGDVGESPALINPATPLAGCEYIHAPHSGM  4198
             ELRGVADVSP QAEKDADAIINALIEGGYIAGDVGESPALINPATPLAGCEYIHAPHSG+
Sbjct  241   ELRGVADVSPEQAEKDADAIINALIEGGYIAGDVGESPALINPATPLAGCEYIHAPHSGL  300

Query  4199  LLNRRELGEWIKPGEVVAEIVDPITDQVTPLVAEFGGVLYARNLMKFATAGMLVVRLAGE  4378
             LLNRRELGEWIKPGEVVAEIVDPITDQVTPLVAEFGGVLYARNLMKFATAGMLVVRLAGE
Sbjct  301   LLNRRELGEWIKPGEVVAEIVDPITDQVTPLVAEFGGVLYARNLMKFATAGMLVVRLAGE  360

Query  4379  NAG  4387
             NAG
Sbjct  361   NAG  363
```

succinylglutamate desuccinylase [Pantoea sp. BL1]

Sequence ID: **WP_045832306.1** Length: 371 Number of Matches: 1

See 1 more title(s)

Range 1: 1 to 363

Score	Expect	Method	Identities	Positives	Gaps	Frame
736 bits(1901)	0.0()	Compositional matrix adjust.	358/363(99%)	360/363(99%)	0/363(0%)	+2

Features:

```
Query  3299  MQQQHHPLLSASLGTQREIVSFHFGTDSQQRVYIQAALHGDELPGMAVAWYLKHKLLALE  3478
             MQQQHHPLLSASLGTQREIVSFHFGTDSQQRVYIQAALHGDELPGMAVAWYLKHKLLALE
Sbjct  1     MQQQHHPLLSASLGTQREIVSFHFGTDSQQRVYIQAALHGDELPGMAVAWYLKHKLLALE  60

Query  3479  SAGQLKSKITLVPVANPLAMGQHWHGSHLGRFHTLSGQDFNRRFPALGDTLAEELAGSLT  3658
             SAGQLKSKITLVPVANPLAMGQHWHGSHLGRFHTLSGQDFNRRFPALGDTLAEELAGSLT
Sbjct  61    SAGQLKSKITLVPVANPLAMGQHWHGSHLGRFHTLSGQDFNRRFPALGDTLAEELAGSLT  120

Query  3659  QSEYENKRLIRDAIDRHYRDRVARTELDAQRFTLMRMASQADLMIDLHCDWDALPHLYTT  3838
             QSEYENKRLIRDAIDRHYRDR+ARTELDAQRFTLMRMASQADLMIDLHCDWDALPHLYTT
Sbjct  121   QSEYENKRLIRDAIDRHYRDRIARTELDAQRFTLMRMASQADLMIDLHCDWDALPHLYTT  180

Query  3839  PHAWQDIEPLARWLGSEVQLLAQISGGEPFDEACCEPWLTLAERFGGEYPMPRGLLPVTL  4018
             PHAWQDIEPLARWLGSEVQLLAQISGGEPFDEACCEPWLTLAERFGGEYPMPRGLLPVTL
Sbjct  181   PHAWQDIEPLARWLGSEVQLLAQISGGEPFDEACCEPWLTLAERFGGEYPMPRGLLPVTL  240

Query  4019  ELRGVADVSPGQAEKDADAIINALIEGGYIAGDVGESPALINPATPLAGCEYIHAPHSGM  4198
             ELRGVADVSP QAE DADAIINALIEGGYIAGDVGESPALINPATPLAGCEYIHAPHSG+
Sbjct  241   ELRGVADVSPEQAENDADAIINALIEGGYIAGDVGESPALINPATPLAGCEYIHAPHSGL  300

Query  4199  LLNRRELGEWIKPGEVVAEIVDPITDQVTPLVAEFGGVLYARNLMKFATAGMLVVRLAGE  4378
             LLNRRELGEWIKPGEVVAEIVDPITDQVTPLV EFGGVLYARNLMKFATAGMLVVRLAGE
Sbjct  301   LLNRRELGEWIKPGEVVAEIVDPITDQVTPLVVEFGGVLYARNLMKFATAGMLVVRLAGE  360

Query  4379  NAG  4387
             NAG
Sbjct  361   NAG  363
```

succinylglutamate desuccinylase [Pantoea sp. SM3]
Sequence ID: **WP_045815228.1** Length: 371 Number of Matches: 1

See 1 more title(s)
Range 1: 1 to 363

Score	Expect	Method	Identities	Positives	Gaps	Frame
736 bits(1899)	0.0()	Compositional matrix adjust.	357/363(98%)	360/363(99%)	0/363(0%)	+2

Features:

```
Query  3299  MQQQHHPLLSASLGTQREIVSFHFGTDSQQRVYIQAALHGDELPGMAVAWYLKHKLLALE  3478
             MQQQHHPLLSASLGTQREIVSFHFGTDSQQRVYIQAALHGDELPGMAVAWYLKHKLLALE
Sbjct  1     MQQQHHPLLSASLGTQREIVSFHFGTDSQQRVYIQAALHGDELPGMAVAWYLKHKLLALE  60

Query  3479  SAGQLKSKITLVPVANPLAMGQHWHGSHLGRFHTLSGQDFNRRFPALGDTLAEELAGSLT  3658
             SAGQLKSKITLVPVANPLAMGQHWHGSHLGRFHTLSGQDFNRRFPALGDTLAEELAGSLT
Sbjct  61    SAGQLKSKITLVPVANPLAMGQHWHGSHLGRFHTLSGQDFNRRFPALGDTLAEELAGSLT  120

Query  3659  QSEYENKRLIRDAIDRHYRDRVARTELDAQRFTLMRMASQADLMIDLHCDWDALPHLYTT  3838
             QSEYENKRLIRDAIDRHYRDR+ARTELDAQRFTLMRMASQADLMIDLHCDWDALPHLYTT
Sbjct  121   QSEYENKRLIRDAIDRHYRDRIARTELDAQRFTLMRMASQADLMIDLHCDWDALPHLYTT  180

Query  3839  PHAWQDIEPLARWLGSEVQLLAQISGGEPFDEACCEPWLTLAERFGGEYPMPRGLLPVTL  4018
             PHAWQDIEPLARWLGSEVQLLAQISGGEPFDEACCEPWLTLAERFGGEYPMPRGLLPVTL
Sbjct  181   PHAWQDIEPLARWLGSEVQLLAQISGGEPFDEACCEPWLTLAERFGGEYPMPRGLLPVTL  240

Query  4019  ELRGVADVSPGQAEKDADAIINALIEGGYIAGDVGESPALINPATPLAGCEYIHAPHSGM  4198
             ELRGVADVSP QAEKDADAIINALIEGGYIAG+ GESPALINPATPLAGCEYIHAPHSG+
Sbjct  241   ELRGVADVSPLQAEKDADAIINALIEGGYIAGETGESPALINPATPLAGCEYIHAPHSGL  300

Query  4199  LLNRRELGEWIKPGEVVAEIVDPITDQVTPLVAEFGGVLYARNLMKFATAGMLVVRLAGE  4378
             LLNRRELGEWIKPGEVVAEIVDPITDQVTPLV EFGGVLYARNLMKFATAGMLVVRLAGE
Sbjct  301   LLNRRELGEWIKPGEVVAEIVDPITDQVTPLVVEFGGVLYARNLMKFATAGMLVVRLAGE  360

Query  4379  NAG  4387
             NAG
Sbjct  361   NAG  363
```

BLAST is a registered trademark of the National Library of Medicine

Support center Mailing list YouTube

- National Library Of Medicine
- National Institutes Of Health
- U.S. Department of Health & Human Services
- USA.gov

NCBI
National Center for Biotechnology Information, U.S. National Library of Medicine 8600 Rockville Pike, Bethesda MD, 20894 USA
Policies and Guidelines | Contact
